# The role of digital health in palliative care for people living with HIV in sub-Saharan Africa: A systematic review

**DOI:** 10.1177/20552076221133707

**Published:** 2022-11-22

**Authors:** Christopher Mwase, Kennedy Nkhoma, Mathew J Allsop

**Affiliations:** 1 Academic Unit of Palliative Care, Leeds Institute of Health Sciences, 4468 University of Leeds, UK; 2 4616 King's College London, UK

**Keywords:** digital health, telemedicine, palliative care, HIV, sub-Saharan Africa

## Abstract

**Background:**

In 2018, 26.6 million people were living with HIV in sub-Saharan Africa. Palliative care services are recommended for people living with HIV at all stages from diagnosis through to end-of-life. However, the provision of palliative care in sub-Saharan Africa is limited, leading to little or no access for the majority of patients. Digital technologies in sub-Saharan Africa present an opportunity to improve access to palliative care for people living with HIV in the region. This review synthesised literature on digital health interventions for palliative care for people living with HIV in sub-Saharan Africa and assessed their effects on patient outcomes.

**Methods:**

Literature searches were conducted in MEDLINE, Embase, PsycINFO and Global Health. Inclusion and exclusion criteria were applied. Two independent reviewers conducted study screening, data extraction and quality appraisal. A narrative synthesis was performed to draw together and report findings across heterogeneous studies. Reporting of this review follows the Preferred Reporting Items for Systematic Review and Meta-Analysis checklist.

**Results:**

Out of 4117 records, 25 studies were included, covering 3592 people living with HIV, across 21 countries. Studies included three randomised controlled trials, three qualitative, three pre- and post-test, two observational, two case series, six cross-sectional and six mixed methods studies. Telemedicine was the most reported digital health intervention, with 12 studies demonstrating the effectiveness of digital health interventions.

**Conclusion:**

Emerging evidence suggests digital health interventions can be effective in facilitating patient-provider communication and health professional decision-making as a part of palliative care for people living with HIV. There is a need for further development and evaluation of digital health interventions alongside determining optimal approaches to their implementation as a part of palliative care provision in sub-Saharan Africa.

## Introduction

In 2018, 37.9 million people were living with HIV (PLWH) globally and 26.6 million (68%) were from sub-Saharan Africa (SSA).^1^ Antiretroviral therapy (ART) has transformed the HIV pandemic into a chronic disease.^2^ There is a need for palliative care for PLWH as it is an integral part of HIV care from diagnosis to end of life.^3^ PLWH have a high prevalence of psychological and physical symptoms, including worry, anxiety, depression, diarrhoea, constipation and insomnia.^4^ When accessed, palliative care can improve patient outcomes across multiple domains.^5^ Palliative care involves the prevention and relief of physical, emotional, social or spiritual suffering associated with any chronic or life-threatening illness, is fundamental to health and human dignity and is a basic human right.^6^ Palliative care is an essential service within universal health coverage^7^; in 2014, a World Health Assembly Resolution called on national governments to carry out actions to develop and strengthen palliative care.^8^

Despite the need for palliative care and its positive impact on patient outcomes, coverage in SSA is greatly below need,^9^ driven by a multitude of factors including unavailability, isolated services, limited funding, lack of inadequate policy and inadequate referral practices.^10^ The application of digital health approaches as health systems strengthening tools has been highlighted through WHO guidance.^11^ The application of information and communication technologies systems can be used to deliver one or more digital health interventions including, for example, systems for client communication, telemedicine, health management information and electronic medical records.^12^ Digital health technologies can be leveraged through existing palliative care models in SSA (see Table 1) to improve access to palliative care by reaching patients in remote areas,^13^ providing e-learning to healthcare providers^14^ and routinely collecting data to inform policy.^15^ SSA is the fastest growing consumer market for mobile phone services with 456 million unique mobile subscribers in 2018 (44% penetration rate) and is estimated to reach 623 million subscribers (50% penetration rate) by 2025.^16^ Digital innovation in SSA is being driven by mobile phones.^16^

**Table 1.** Three models of palliative care in SSA.

Models of palliative care in SSA as reported in Downing et al. (2015)^69^	Description of model
Community	Healthcare workers deliver palliative care in the homes of patients^76^ or at a selected location within the community^77^ and make referrals to the district level where appropriate.^69^ Caregivers also provide palliative care within the home of the patient.^19^
District hospital	Palliative care is provided to both outpatients and in-patients referred from the community level for further care or from specialists for continued management.^78,79^
Specialist	Palliative care is usually provided by specialist teams or doctors at tertiary hospitals who receive referrals from facilities at lower or a similar level.^78,80^

SSA: sub-Saharan Africa.

The Essential Palliative Care Package for Universal Health Coverage^17^ highlights digital health as an approach to increasing access to palliative care services. A systematic review^18^ of literature up until 2015 found that in SSA, mobile phones are starting to be used to improve access to palliative care by enabling patients to communicate with providers,^19–21^ to encourage patients to adhere to appointments^22^ and for health provider education.^23^ However, the evidence underpinning digital health for palliative care in SSA is still underdeveloped.^24^ Emerging evidence suggests the potential of digital technologies to support PLWH with self-management, medication adherence and facilitating communication with health professionals.^25,26^ However, to date, there have been no reviews with a focus on digital health approaches for palliative care among PLWH in SSA. This review addresses the gap by synthesising existing literature to date and reporting on the effectiveness of digital health interventions on patient outcomes.

## Methodology

### Objectives

This review addresses two questions: (a) What digital health interventions are being used to provide palliative care to PLWH in SSA? (b) What is the effectiveness of identified digital health interventions on patient outcomes? A population, intervention, comparator, outcomes and study (PICOS) framework was used to structure the review. This included people living with HIV in SSA (population), digital health (intervention/exposure), usual care or no comparator (comparison), physical, psychological, social or spiritual symptoms, a focus on quality of life, patient satisfaction with palliative care services, and other patient outcomes relevant to palliative care (outcomes). This review was conducted according to the Preferred Reporting Items for Systematic Reviews and Meta-Analyses (PRISMA).^27^ A full protocol is registered in PROSPERO (reference number: CRD42020182695).

### Eligibility

Included studies included PLWH, a digital health intervention (defined broadly as the use of information and communications technology in support of health and health-related fields^28^) involving palliative care patients and/or providers, reported patient outcomes and were conducted in SSA. Studies with or without comparators were also included, alongside studies where digital health was part of a combined intervention package. Any study design or setting (e.g. community and hospital) was included. Articles published in any language were eligible for inclusion. Studies were excluded if they had patients with diseases other than HIV, no digital health intervention, did not report patient outcomes and were conducted outside SSA. Studies with digital health interventions used in HIV prevention, testing, ART adherence and viral load monitoring were excluded except when the intervention was explicitly used to support palliative care for PLWH. Studies were also excluded if they did not report primary data.

### Search of studies

Search strategies (see Supplementary Material 1) were developed with guidance from information specialists at the university of the lead author. Literature searches were conducted in MEDLINE, Embase, PsycINFO and Global Health on 22 May 2020 with no limit on publication date. The search strategies were adapted with relevant Boolean operators and search characters for each database. A combination of search terms for ‘HIV’, ‘Digital Health’ and ‘sub-Saharan Africa’ was used to capture all digital health literature in HIV care for the SSA region. Combinations of MeSH terms and keywords were used to search the databases. EndNote X9 was used to store and manage the references exported from the databases alongside identifying and removing duplicate citations.

### Study selection and data collection

**Figure 1.**
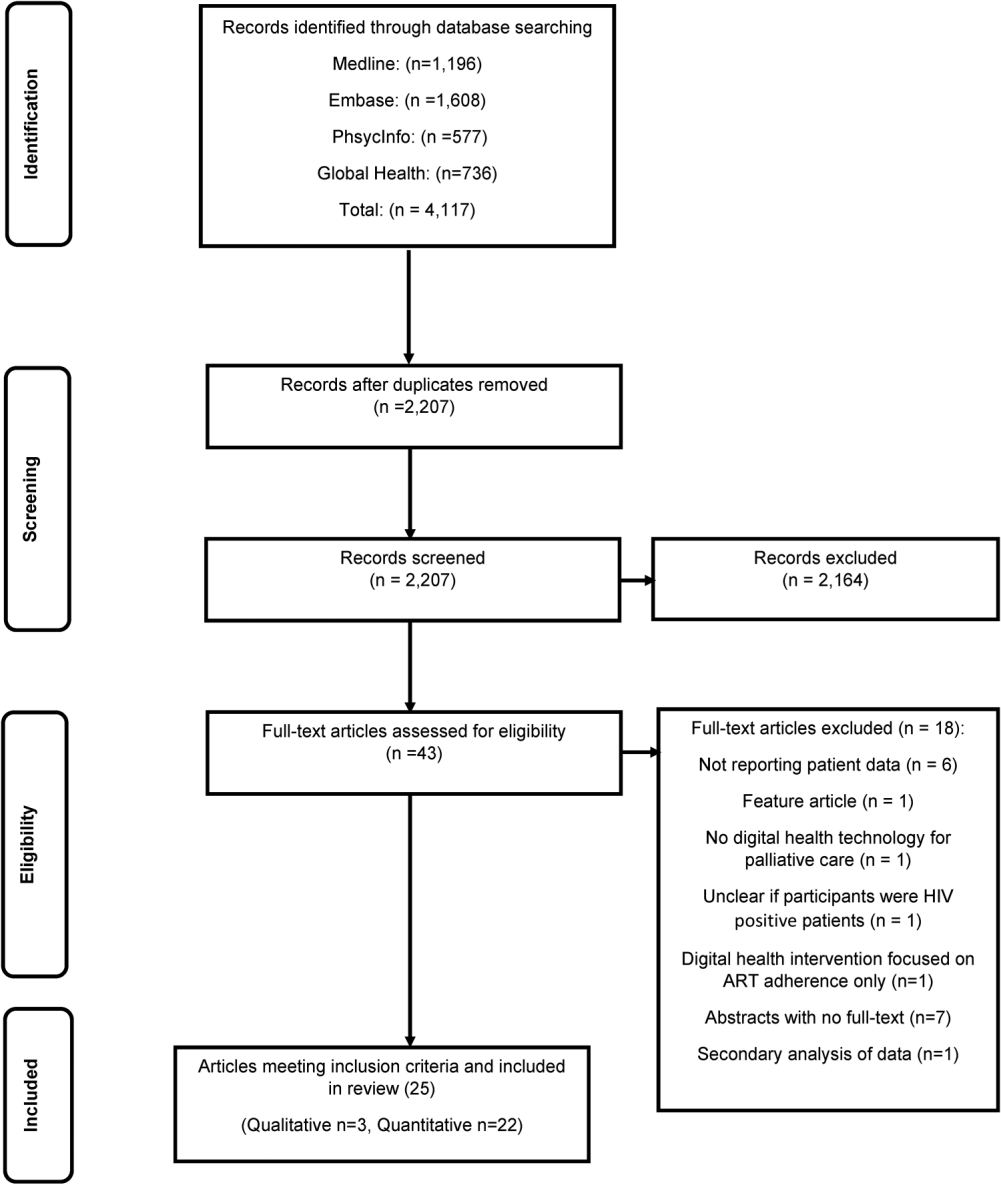
PRISMA flow chart for the systematic review.

Two reviewers (CM and MJA) independently reviewed titles and abstracts against the inclusion and exclusion criteria. Full-text articles were sought for the included studies and their content was assessed against the inclusion and exclusion criteria. Any disagreements between the two reviewers were resolved through discussion. KN reviewed the final list of the selected studies. Figure 1 presents the PRISMA flow diagram^27^ for this review. A data extraction form was developed based on the Cochrane Collaboration^29^ and converted into an Excel spreadsheet. The spreadsheet was used to extract and store data from the included studies (see Supplementary Material 2). CM extracted the data. The data were checked by MJA and KN. A WHO template for Classification of Digital Health Interventions version 1.0^30^ was used to categorise digital health interventions. A Template for Intervention Description and Replication (TIDieR)^31^ was used to extract details of each intervention included in the review (see Supplementary Material 3).

### Risk of bias assessment

The Mixed Methods Appraisal Tool^32^ was used to assess the risk of bias for the individual studies (see Supplementary Material 4). CM appraised the studies and MJA reviewed the appraisal. Any discrepancies were resolved through discussion. KN cross-checked the final appraisal.

### Statistical analysis

Due to the heterogeneity of the included studies, a descriptive synthesis^33^ was used to summarise the included studies. This was followed by a classification of the digital health interventions by the WHO taxonomy^30^ and descriptions of the interventions according to the TIDieR checklist.^31^ A framework for the development of complex interventions^34^ was also used to describe the stages of development of digital health interventions described in included studies.

## Results

In total, 4117 records were identified. After de-duplication, 2207 articles remained. The articles were screened and a full-text review carried out for 43 articles. Following full-text review, 25 articles were included in the review. Included studies used quantitative (*n*  =  22) and qualitative (*n*  =  3) study approaches (see Figure 1).

### Characteristics of included studies

Studies included a total of 3592 PLWH. Sample sizes of the studies varied from 10^35^ to 2458.^36^ Included studies were conducted in 21 countries: South Africa (*n*  =  5), Kenya (*n*  =  4), Botswana (*n*  =  3),^37–39^ Nigeria (*n*  =  3),^40–42^ Uganda (*n*  =  2),^43,44^ Gabon (*n*  =  1),^35^ Ghana (*n*  =  1),^45^ Lesotho (*n*  =  1),^46^ Malawi (*n*  =  1), Zimbabwe (*n*  =  1)^47^ and multiple countries (*n*  =  3).^36,48,49^ Studies included three randomised controlled trials (RCTs),^41,43,50^ three qualitative studies,^47,51,52^ three pre- and post-test studies,^42,44,53^ two observational studies,^49,54^ two case series,^35,36^ six cross-sectional studies^37–39,48,55,56^ and six mixed method studies.^40,45,46,57–59^ Most studies were published in 2019 (*n*  =  6),^48,49,53–55,57^ followed by 2018 (*n*  =  4),^36,40,56,59^ 2015 (*n*  =  4)^41,44,50,51^ and 2017 (*n*  =  3).^45,46,52^ Table 2 summarises the characteristics of the included studies, and Table 3 provides further details of study characteristics. All data extracted from the studies are available in Supplementary Material 2.

**Table 2.** Summary of study characteristics.

		Number of studies (%)	Study designs	Range of sample size	Number of PLWH (%)
All studies		25 (100%)			3592 (100%)
Country of origin	Botswana	3 (12%)	Cross-sectional	75–99	175 (4.9%)
	Gabon	1 (4%)	Case series	10	10 (0.3%)
	Ghana	1 (4%)	Mixed methods	50^a^	22^b^ (0.6%)
	Kenya	4 (16%)	Qualitative, pre- and post-test, cross-sectional and mixed methods	45–263	417 (11.6%)
	Lesotho	1 (4%)	Mixed methods	835	633^c^ (17.6%)
	Malawi	1 (4%)	Observational	194	183^d^ (5.1%)
	Nigeria	3 (12%)	Pre- and post-test, RCT and mixed methods	19–132	192 (5.3%)
	South Africa	5 (20%)	RCT, cross-sectional, mixed methods and qualitative	37–129	377 (10.5%)
	Uganda	2 (8%)	RCT and pre- and post-test	33–60	60^e^ (1.7%)
	Zimbabwe	1 (4%)	Qualitative	12	12 (0.3%)
	Multicountry (Côte d’Ivoire, Burkina Faso, Togo)	1 (4%)	Cross-sectional	1131	1131 (31.5%)
	Multicountry (Mozambique, Burundi, Swaziland, Togo, Central African Republic, Guinea Conakry, Democratic Republic of the Congo, Nigeria, Malawi, Tanzania, Cameroon, Kenya)	1 (4%)	Case series	2458	134^f^ (3.7%)
	Multicountry (South Africa, Malawi, Uganda, Zimbabwe)	1 (4%)	Observational	615	246^g^ (6.8%)

PLHIV: people living with HIV.

^a^ The number of key informants who were also participants in the study is not reported.

^b^ Only 22 were PLHIV. The study also had 21 men who have sex with men (MSM) and 14 sex workers.

^c^ Excludes 202 treatment supporters.

^d^ Excludes 11 medical personnel.

^e^ The first study had 60 participants, and 33 participants of the second study were drawn from the first study.

^f^ 2458 teleconsultations were done, and only 5.5% (*N*  =  134) were for neurological issues, which was the focus of the study.

^g^ The study had 615 participants. Only 246 were HIV-infected children.

**Table 3.** Detailed characteristics of included studies.

No. in Table 3	First author and year	Report title	Population description (where the participants are drawn)	Setting of study	Country	Aim of study	Study design	Mean/median age	Gender	Results	Conclusions
1	Azfar, 2014	Reliability and validity of mobile tele-dermatology in human immunodeficiency virus-positive patients in Botswana: A pilot study	HIV-positive patients in medical and oncologic wards, dermatologic clinic, infectious disease clinic, private primary care clinic and outpatient clinics (*n* = 76)	Oncologic wards, dermatologic clinic, infectious disease clinic, private primary care clinic and outpatient clinics	Botswana	To determine whether the use of mobile tele-dermatology technology in HIV-positive patients in Gaborone, Botswana, was reliable and produced valid assessments compared with face-to-face dermatologic consultations.	Cross-sectional, quantitative, assessed effectiveness of the digital health intervention	Median age was 39 years	43 (57%) were female	Agreement between the face-to-face dermatologist and the remote reviewers for the primary diagnosis ranged from 47% for evaluator 2 (κ: 0.41; 95% CI: 0.31 to 0.52) to 57% for evaluator 4 (0.51; 0.41 to 0.61). Agreement between the face-to-face dermatologist and the remote reviewers on how to treat the patient's primary diagnosis ranged from 32% for evaluator 2 (κ: 0.08; 95% CI: 0.02 to 0.15) to 51% for evaluator 4 (0.12; 0.01 to 0.23).	First attempt at validating mobile tele-dermatology in this practice setting. Much work is needed to optimise and validate the use of this technology on a larger scale in this population.
2	Azfar, 2011	HIV-positive patients in Botswana state that mobile tele-dermatology is an acceptable method for receiving dermatology care	HIV-positive patients in medical and oncologic wards, dermatologic clinic, infectious disease clinic, private primary care clinic and outpatient clinics (*n* = 75)	Oncologic wards, dermatologic clinic, infectious disease clinic, private primary care clinic and outpatient clinics	Botswana	To determine if patients infected with HIV in resource-limited settings such as southern Africa find the use of mobile phones acceptable for collecting their health information and would be willing to receive skin care through this method.	Cross-sectional, quantitative, neither assessed efficacy nor effectiveness of the digital health intervention	Median age was 39 years	34 (44%) were male	The majority of patients stated that time (76%), costs (57%) and distance (41%) were the major barriers in seeking medical care for their skin conditions. If privacy was guaranteed, 99% of patients reported that they would be completely comfortable with a mobile tele-dermatology consultation.	Overall, mobile tele-dermatology consultations were well accepted by HIV-positive patients with mucocutaneous conditions in Botswana.
											
3	Boivin, 2010	A pilot study of the neuropsychological benefits of computerised cognitive rehabilitation in Ugandan children with HIV	HIV-positive children provided with home-based healthcare (*n* = 60)	Community	Uganda	To establish the feasibility and gather preliminary evidence for the efficacy of computerised cognitive rehabilitation therapy (CCRT) with African children in a low-resource setting, as a proof-of-concept for the potential of such an intervention for HIV-affected children globally.	Randomised controlled trial, quantitative, assessed the efficacy of the digital health intervention	Mean age of 9.36 years in control group and 10.34 years in intervention group	15 (53.6%) females in control group and 21 (65.6%) in intervention group	Highly significant differences on both maze learning (mean difference = -0.07, SE = 0.02, *p* = .001) and card detection speed (mean difference = 0.07, SE = 0.02, *p* = .01). For the adjusted intervention effects, highly significant intervention effects are found for maze learning (group effect = -0.06, SE = 0.02, *p* < .001) and card detection speed (group effect = 0.06, SE = 0.02, *p* = .02).	CCRT was feasible with the study population and improved maze learning and attention on a detection task.
4	Dulli, 2018	An online support group intervention for adolescents living with HIV (ALHIV) in Nigeria: A pre-post-test study	HIV-positive adolescents attending clinics (*n* = 41)	Clinic and community	Nigeria	To develop and test the feasibility and acceptability of a structured support group intervention—SMART (Social Media to promote Adherence and Retention in Treatment) Connections—which is delivered through a social media platform to improve retention in HIV health services and ART adherence among ALHIVs aged 15–19 years in peri-urban southern Nigeria.	Mixed-methods, quantitative, neither assessed efficacy nor effectiveness of the digital health intervention	Median age was 17 years	22 (53%) were female	Most participants who completed the endpoint questionnaire (34 of 35) participated in the intervention sessions. Just over half of the 16 in-depth interview respondents mentioned occasional problems with charging their phone or running out of data. About one-third of IDI respondents mentioned issues with cellular networks.	This feasibility study demonstrated that an online support group intervention was both feasible and acceptable among ALHIVs in southern Nigeria.
											
5	Giordani, 2015	Designing and evaluating brain powered games (BPG) for cognitive training and rehabilitation in at-risk African children	Controls from Boivin, 2010 (*n* = 33)	Clinic and community	Uganda	To develop a computer-based training platform, BPG, suitable for use with children within a rural, sub-Saharan Africa setting and then complete an initial field trial with that program.	Pre-and post-test, quantitative, assessed effectiveness of the digital health intervention	Mean age was 8.55 years	20 (61%) were female	Results comparing the ‘control period’ against post-BPG show that there was a large effect size (1.09) for the CogState Groton Maze Learning Test Learning Score (correct moves per second) (*p* < .01). There were also large effect sizes (1.29) for CogState Groton Maze Learning Test Chase Score (correct moves per second) (*p* < .01) and (0.79) for test of variables of attention (TOVA) response time (ms) (*p* < .01).	Results of this study demonstrate that it is possible to create a computerised, game-based training platform that is appropriate for children in sub-Saharan Africa regions that are rural and poor in resources and experience with Western-based materials.
6	Graham, 2015	Development and pilot testing of an intervention to promote care engagement and adherence among HIV-positive Kenyan MSM	ART experienced (*n* = 20) and ART naïve men who have sex with men (MSM) (*n* = 20) Local providers serving MSM clients (*n* = 29)	Clinic and community	Kenya	To design a targeted, culturally appropriate intervention to promote men's care engagement and ART adherence, and to describe the safety, feasibility, and acceptability of this intervention based upon a small pilot study.	Qualitative, neither assessed efficacy nor effectiveness of the digital health intervention	Intervention development: Median age of providers was 36 (28–59) years Median age of MSM was 31 (19–51) years Intervention pilot: MSM age range was 24–42 years	14 (48.3%) of the providers asked about intervention development were male	Several MSM were intrigued by Internet-based health promotion, pointing out the appeal of maintaining anonymity. However, because Internet access is uncommon, training and improved access would be needed to make this approach feasible. Of MSM asked about feasibility of a telephone hotline, several favoured the idea but voiced reservations about potential misuse. A few respondents thought that education about the hotline's purpose and staff would be needed, in order to assure men of confidentiality. Providers suggested that in addition to sites tailored to MSM patient needs, there should be sites to provide information and other services to men's family and friends, ‘‘who need to be able to live with them and support them, and the first step is in understanding them’.’	An adherence intervention targeting HIV-positive MSM living in an African setting was developed and it was later used in an RCT.
7	Hacking, 2019	Peer mentorship via mobile phones for newly diagnosed HIV-positive youths in clinic care in Khayelitsha, South Africa: Mixed methods study	Newly diagnosed HIV positive youth at ART clinics (*n* = 110) Stable youth in HIV care (*n* = 19)	Clinic	South Africa	To determine if peer-to-peer mentorship, specifically between newly diagnosed HIV-positive youths and HIV-positive youths stable in care, could be successfully implemented using mobile phones as the primary means of communication.	Mixed methods, quantitative, assessed effectiveness of the digital health intervention	Intervention group median age: 20 years 5 months control group median age: 22 years 7 months	Intervention group: 38 (95%) were female Control group: 64 (91%) were female	Mentees had increased antiretroviral initiation (28/35, 80% vs 30/70, 42% in matched controls) and viral load completion (28/35, 80% vs 32/70, 45%). No differences were found in viral load suppression. No differences were found in retention in care (RIC) at 6 or 12 months.	A peer-to-peer navigator program for newly diagnosed HIV-positive youths, conducted via mobile phone, might have increased linkage to ART care but had no impact on retention in care.
8	Heerden, 2017	Perceived mHealth barriers and benefits for home-based HIV testing and counselling and other care: Qualitative findings from health officials, community health workers, and persons living with HIV in South Africa	Home-based HIV testing and counselling (HTC) field staff (*n* = 10) Community health workers (CHWs) (*n* = 12) Persons living with HIV (PLH) (*n* = 10) Key informants (KI): familiar with electronic patient tracking systems (*n* = 5)	Research offices and key informant places of work	South Africa	To inform the development of a mobile platform to be integrated into a home-based HTC program to assist CHWs linking PLWH to HIV care.	Qualitative, neither assessed efficacy nor effectiveness of the digital health intervention	PLH: median = 25.5 years old; range = 19–41 Staff: median = 31.5; range = 26–42 CHWs: median = 30, range = 19–58 KI: median = 45; range = 42–58	PLH = 10 (100%) were female Field staff = 4 males, 6 females CHWs = 12 (100%) were female KI = 1 man, 4 women	All stakeholders brought up a lack of communication in sharing patient health information between clinics, between clinics and CHWs, and between clinics and patients as major barriers to care that mHealth can address. CHWs need better patient information from clinics in terms of physical location and health status to plan visitation routes and address patient needs. CHWs perceive that communication barriers create distrust towards them by clinic staff. PLH wanted automated appointment and medication reminders. KI. saw mHealth as a way to improve health information transfer to government officials to better allocate healthcare resources.	All stakeholders provided useful information towards the development of mHealth systems.
9	Henwood, 2016	Acceptability and use of a virtual support group for HIV-positive youth in Khayelitsha, Cape Town using the MXit social networking platform	Patients attending an HIV care clinic adapted to the needs of young people aged 12–25 years (*n* = 90)	Clinic	South Africa	To evaluate the acceptability and uptake of the MXit chat-room for HIV-positive youth.	Mixed methods, quantitative, neither assessed efficacy nor effectiveness of the digital health intervention	58% of the respondents were between 23 and 25 years	63% were female	Thirty-four percentage used the chat-room at least once, 20% had visited the chatroom in the past month. Twenty-nine percentage (29%) had used MXit to have private conversations with other club members Fifty-seven percentage (57%) used the chat-room to get advice, and 84% of all respondents felt that offering a service outside the youth club meetings was important and would like to see one to continue.	Reported usage of the MXit chat-room was low, but participants indicated acceptance of the programme and their desire to interact with their peers through social media.
10	Hirsch-Moverman, 2017	Using mHealth for HIV/TB treatment support in Lesotho: Enhancing patient–provider communication in the START study	TB/HIV co-infected patients at six health facilities that received a combination intervention package (CIP) as part of the START trial (*n* = 633) Treatment supporters (*n* = 202)	Health facilities	Lesotho	To describe the use and acceptability of the mHealth component of the START study intervention at the 6 CIP health facilities, where the intervention was implemented.	Mixed methods, quantitative, neither assessed efficacy nor effectiveness of the digital health intervention	Mean age of 38.1 years	43.3% were female	Phone calls, above and beyond SMS messages, empowered patients to communicate with their health care providers and treatment supporters in a timely manner, without incurring a personal cost Patients said they more frequently called their provider to report a side effect, seek advice, or inform their clinic about potential delays to a clinic appointment. They also felt more inclined to call and request assistance from their treatment supporters. Health care provider participants reported receiving sufficient training and technical support to partake in phone related study activities. They expressed support for the mHealth component of the intervention as it facilitated communication between patients, treatment supporters, and the various cadres of providers engaged in the CIP.	The mHealth intervention for HIV/TB treatment support in Lesotho was found to be a low-technology, user-friendly intervention, which was acceptable to patients and health care providers.
											
11	Ishola, 2015	The use of mobile phones to deliver acceptance and commitment therapy (ACT) in the prevention of mother–child HIV transmission in Nigeria	HIV-positive pregnant women attending four PMTCT centres in south-western Nigeria. (*n* = 132)	PMTCT centres	Nigeria	To develop, implement and evaluate ACT in prevention of mother to child HIV transmission (PMTCT) programmes in Nigeria using weekly mobile phone messages with the aim of increasing psychological flexibility of HIV-positive pregnant women in Nigeria.	Randomised controlled trial (Solomon four-group design), Quantitative, assessed the efficacy of the digital health intervention	Mean age was 31.6 years	All participants were female	An independent samples t-test was used to compare the pre- and post-test score differences between Group 1 (mean (M) = 3.6, standard deviation (SD) = 8.9) and Group 2 (M = 4.9, SD = 11.1). There was a significant psychological flexibility improvement in participants following ACT intervention (*t* = 3.4, *p* < .001). The ANOVA (*F*(1,33) = 19.2, *p* < .001) found a significant interaction between the intervention and pre-test factors suggesting pre-test sensitisation being present.	The introduction of mobile phone-based ACT may result in greater psychological flexibility in women diagnosed with HIV.
12	Ivanova, 2019	Evaluation of the ELIMIKA pilot project: improving ART adherence among HIV-positive youth using an eHealth intervention in Mombasa, Kenya	HIV-positive youth attending coast provincial general hospital comprehensive care clinic (CCC) and family care clinic (FCC) in Mombasa, Kenya (*n* = 90)	Clinic	Kenya	To evaluate the usability and the effectiveness of a pilot digital peer support system in improving HIV/ART knowledge, perceived importance of adherence, perceived self-efficacy in adhering and future intentions towards adherence.	Pre- and post-test, quantitative, assessed effectiveness of the digital health intervention	Mean age of 18.4 years	45 (55.6%) were female	The majority (95%) stated their intentions (agree very much and agree) to use the ELIMIKA website again and 87% would recommend it to others. Many young people needed help with using ELIMIKA platform (63%). In general, total knowledge scores improved by 0.3 points; however, this effect was not found to be statistically significant (Wilcoxon signed ranks test – 0.26).	Despite a lack of effectiveness of this intervention, this study provides valuable information on feasibility and challenges of the digital platform in local Kenyan context that might support further research in this area.
13	Janssen, 2013	'Remote FASH’ tele-sonography – A novel tool to assist diagnosing HIV-associated extrapulmonary tuberculosis in remote areas	HIV-positive patients attending an HIV outpatient clinic at the general hospital in Lambaréné, Gabon (*n* = 10)	HIV outpatient clinic	Gabon	To explore the possibility to use remote ultrasound in the diagnostic work-up for HIV-associated EPTB, and potentially for other (intra-abdominal) pathologies.	Case series, quantitative, neither assessed efficacy nor effectiveness of the digital health intervention	Mean age was 38.3 years	7 females and 3 males	Extra-pulmonary TB was diagnosed in 4 of the 10 participants. One of the four cases had already been diagnosed with TB through sputum microscopy. In the other 3 cases sputum microscopy and chest x-ray were negative. The 6 negative patients did not develop any unmasking TB.	The case series shows possibilities to use remote ultrasound in the diagnostic work-up for HIV-associated EPTB, and potentially for other (intra-abdominal) pathologies.
14	John, 2016	Enhancing self-care, adjustment and engagement through mobile phones in youth with HIV	Non-disclosed adolescents and young adults (15–29 years) living with HIV in Calabar, South-South region of Nigeria (*n* = 19)	Community	Nigeria	Evaluating the effectiveness of mobile phones in enhancing self-care, adjustment and engagement in non-disclosed youth living with HIV in Nigeria.	Pre- and post-test, quantitative, assessed effectiveness of the digital health intervention	The age range was 15–29 years	12 (62.3%) were male	Mean scores on self-care capacity changed from 21.6 (pre-test) to 45.8 (*p* < .001 at post-test 1) and 51.5 (*p* = .02 at post-test 2). Psychological adjustment scores increased from 25.6 to 58.9 (*p* < .001 at post-test 1) and 103.3 (*p* < .001 at post-test 2), especially in the maintenance of a sense of self-worth.	Mobile phones enhance self-care and psychological adjustment and facilitate engagement in non-disclosed youth living with HIV.
15	Kaminski, 2019	Clinical stage of acquired immunodeficiency syndrome in HIV-positive patients impacts the quality of the touch electrocardiogram (ECG) recordings	Patients with different clinical stages of established HIV infection at two outpatient clinics in Kenya. (*n* = 263)	Outpatient clinic	Kenya	To investigate the quality of the ECG signal acquired by a touch ECG device (Kardia) in patients with different clinical stages of established HIV infection	Cross-sectional (prospective), quantitative assessed effectiveness of the digital health intervention	Median age was 46 (39–53) years	203 (77%) were female	There was a significant clinical difference between patients with readable and unreadable ECG based on clinical staging of HIV infection (p < 0.0001) The univariate logistics regression showed that an increase of the World Health Organisation AIDS Clinical Staging (WACS) score by 1 was associated with a significantly higher risk for acquiring an unreadable ECG by Kardia device both in the analysis unadjusted (OR: 1.96; 95% CI: 1.46–2.62; p < .0001) and adjusted (OR: 1.87; 95% CI: 1.38–2.53; p < 0.0001) to patient's’ age, sex, body mass index and time since HIV diagnosis.	Preliminary results of this study will serve as a starting point to a comprehensive prospective ECG study of the HIV patients.
											
16	Leone, 2018	Tele-neurology in sub-Saharan Africa: Experience from a long-lasting HIV/AIDS health program (DREAM)	HIV-positive and HIV-negative patients attending disease relief through excellent and advanced means (DREAM) centres in sub-Saharan Africa (*n* = 2458)	Health centres	Mozambique, Burundi, Swaziland, Togo, Central African Republic, Guinea Conakry, Democratic Republic of the Congo, Nigeria, Malawi, Tanzania, Cameroon, Kenya	To report on the preliminary results of a tele-neurology system in SSA who took advantage of local health-workers education program in neurology and a long-lasting relationship with the external advisers.	Case series, quantitative, neither assessed efficacy nor effectiveness of the digital health intervention	Mean age was 22.8 (25–66) years	61% of the neurological patients were female	Most frequent neurological diagnoses ranged from pain (∼2%) to epilepsia (∼15%) In 72% of the cases the same question for the neurologist was also for at least another specialist, that is, the same question to more than one specialist: cardiology (20%), radiology (18.3%), paediatry (16.7%), internal medicine (15%), orthopaedic (10%), infectivology (10%), angiology (10%).	These preliminary findings show that in a setting where health care personnel receive specific education and training, tele-neurology has the potential to become a powerful tool in fighting the double burden of HIV and neurologic disorders in sub-Saharan Africa.
17	Lepère, 2019	Exploring the patterns of use and acceptability of mobile phones among people living with HIV to improve care and treatment: Cross-sectional study in three Francophone West African Countries	Patients attending HIV clinics at 6 reference centres in Côte d’Ivoire, Burkina Faso, and Togo (*n* = 1131)	HIV clinics	Côte d’Ivoire, Burkina Faso, and Togo	To explore the social and financial acceptability, which has not been reported yet in Francophone Africa, and the feasibility of an mHealth intervention for the improvement of care among PLHIV in West Africa.	Cross-sectional, quantitative, neither assessed efficacy nor effectiveness of the digital health intervention	Median age was 44 (38–51)years	861 (76.1%) were female	Overall, the rate of mobile phone possession was 97.9% (*n* = 1107), with a small difference between countries (98.8%, 95.6%, and 97.9% for Côte d’Ivoire, Burkina Faso, and Togo, respectively; *p* = .01). The overall rate of mHealth acceptability (patients’ acceptability of receiving text messages or phone calls from their physicians) was 98.8% with no variation by country (98.3% in Côte d’Ivoire, 99.1% in Togo, and 100.0% in Burkina Faso; *p * = .08).	Based on a considerable sample size, this study demonstrated that mobile technology is accessible in francophone low-income countries and could be used as a tool to improve the quality of HIV care and treatment.
18	McHenry, 2018	Tablet-based disclosure counselling for HIV-infected children, adolescents, and their caregivers: A pilot study	Clinical officers, nurses and social support staff (clinical providers) who were providing HIV care to adolescents at three clinics in Kenya (*n* = 21) Adolescents receiving HIV care from the clinical providers (*n* = 24)	HIV clinics	Kenya	To evaluate clinical providers’ perceptions and experiences of using tablet computers loaded with multimedia resources for disclosure related counselling with HIV-infected adolescents and their caregivers at several HIV clinics in western Kenya. Additionally, to solicit perspectives from adolescents attending those clinics regarding their experiences with the tablets.	Mixed methods (longitudinal), quantitative, neither assessed efficacy nor effectiveness of the digital health intervention	Not provided for the clinical providers, adolescents’ mean age was 13.8 years	13 (62%) of the providers were female not provided for the adolescents	After tablet initiation, monthly follow-up surveys indicated tablets were used during 75% or more of clinic encounters by 67% (14/21) of providers 1 month after tablet distribution and 85% (18/21) at the end of the study. The adolescents described that the narrative videos helped them feel like they were not alone in their HIV diagnosis, which they described as an improvement of mood and mental health.	Tablet computers with resources for disclosure are an acceptable resource for clinical providers who play a role in HIV disclosure for HIV-infected children and adolescents. The adolescents viewed the tablet computers positively and reported improvements in medication adherence and mood resulting from this intervention.
19	Quinley, 2011	Use of mobile telemedicine for cervical cancer screening	HIV-positive women attending an ART clinic (*n* = 99)	HIV clinic	Botswana	To determine whether mobile telemedicine is safe and effective for cervical cancer screening when employed as an adjunct to visual inspection with acetic acid (VIA).	Cross-sectional, quantitative, assessed effectiveness of the digital health intervention	Median age was 34 years	100% were female	Of all the PIA results determined to be negative by the expert, 89% were also considered negative by the nurses. In addition, 82% of the positive expert PIA readings were also determined to be positive by nurse PIA. Based on the kappa statistic (0.71), the nurses and expert agreed more often than would be expected by chance (*p* < .001) The PIA results of Nurse 1 agreed with those of the expert in 69% of cases, and those of Nurse 2 agreed with the expert in 77% of cases. Both Nurse 3 and Nurse 4 agreed with the expert in every PIA diagnosis, yielding a diagnostic concordance of 100%.	Telemedicine performed with an appropriate mobile camera phone has the advantage of not requiring Internet connections or an electricity supply and allows images to be transmitted immediately. This provides the opportunity for evaluation by a remote expert while the patient is still in the clinic.
20	Robbins, 2018	A mobile app to screen for neurocognitive impairment (NCI): Preliminary validation of neuroscreen among HIV-infected South African adults	Participants were drawn from a larger randomised controlled trial (RCT) of a multimedia, laptop-based, lay health worker-delivered ART-readiness intervention for ART initiators (known as Masivukeni or ‘Let's Wake Up’) conducted in Cape Town, South Africa (*n* = 102)	Clinics	South Africa	To evaluate the ability of the lay health worker administered NeuroScreen to detect NCI, as defined by a gold standard neuropsychological test battery.	Cross-sectional, quantitative assessed effectiveness of the digital health intervention	Mean age was 33.31 years	83 (81%) were female	Gold-standard HIV neuropsychological battery performance: The mean global deficit score (GDS) was 0.36 (SD 0.40) and 26.5% (27/102) had NCI using a GDS of 0.5 or greater to indicate impairment. Sensitivity and specificity: NeuroScreen Total Score 1 (Sum of All Tests): Using the logistic model with the first NeuroScreen total score adjusted for age, education, and sex to predict the gold standard NCI in the receiver operating characteristic (ROC) analysis, the AUC was 0.86 (95% CI: 0.78–0.94. The Youden index NeuroScreen predicted NCI cut-score of 0.21 maximised sensitivity at 81.48% (95% CI: 61.92%–93.70%) and specificity at 74.67% (95% CI: 63.30%–84.01%). The PPV was 53.66% and the NPV was 91.80%. Using this cut-score yielded 19 false positives and 5 false negatives. The mean completion time for all the tests was 23.88 (SD 6.21) minutes.	This study provides evidence that NeuroScreen, has clinically useful psychometric properties to detect NCI when administered by lay health workers. Taking advantage of mobile platforms and automating many components of the neurocognitive testing process may help to make testing more accurate, efficient, affordable and accessible to those who need testing, especially in resource-limited settings.
21	Robbins, 2015	Enhancing lay counsellor capacity to improve patient outcomes with multimedia technology	Patients attending a City of Cape Town Department of Health primary health clinic that provides HIV care and ART near a large township. (*n* = 65)	Primary care (clinic)	South Africa	To examine medication adherence and key psychosocial outcomes among non-adherent South African HIV-positive patients, on antiretroviral therapy (ART) who were randomised to receive either Masivukeni (a digital health intervention) or standard of care (SOC) counselling for ART non-adherence.	Randomised controlled trial, quantitative, assessed efficacy of the digital health intervention	Mean age was 38.46 years in both control and intervention groups	66% were female in control group 67% were female in intervention group	Participants in Masivukeni reported significantly more positive attitudes towards disclosure at post-intervention than participants in the SOC group (*p* = .04). The Masivukeni group reported significantly more medication specific social support to take their ARVs at post-intervention assessment (*p* = .02).	Masivukeni can maximise standardisation of intervention delivery, even by counsellors with varying backgrounds, training, and capacity, thus taking full advantage of the potential of task-shifting in the health care system.
22	Ruiseñor-Escudero, 2019	Building capacity in neurodevelopment (ND) assessment of children in sub-Saharan Africa: A quality assurance (QA)model to implement standardised neurodevelopment testing	Children from 4 sub-Saharan African countries aged 5–11 years; (1) HIV-infected, (2) HIV exposed (perinatally) but uninfected, and (3) HIV unexposed and uninfected infants (*n* = 615) Neurocognitive development evaluators (*n* = 15) Supervisors (*n* = 10)	Multi-site in 4 sub-Saharan African countries	South Africa, Malawi, Uganda and Zimbabwe	To describe and discuss how QA model for ND testing can be used across settings and with personnel of varying experience and backgrounds.	Observational, quantitative, assessed effectiveness of the digital health intervention	Mean age of children across sites ranged from 6.6 to 8.0 years	Percentage of males across sites ranged from 39–55%	The mean total score at the beginning of the evaluation (e.g. evaluator's first video reviewed by the QA centre) was 161 (range:118–180), while the mean total score over the 10 months duration of QA centre supervision was 178 (range: 175–180). At the last video scoring conducted, evaluators on average had a mean total score of 165 (range: 140–180). Rubric scores were considered sufficient (e.g. mean score >70% of maximum score) and none of the evaluators were terminated. Test completion rates were high across all sites; 99% of participants (*n* = 611) evaluated at baseline completing the KABC-II. There were few instances of invalid tests and few barriers to test completion (e.g. disruptive behaviours in the child such as crying or refusing to follow instructions); at each assessment time point, 97% or more of the KABC-II across sites was considered to be valid by the supervisor.	Use of the QualiND model across six study sites in sub-Saharan Africa proved to be an extremely useful framework for the implementation of a standardized test of neurodevelopment as part of a large-scale research study.
23	Schwab, 2019	Use of remote radiology support for training and quality assurance in the ‘focused assessment with sonography for HIV-associated tuberculosis’: A pilot program in Malawi	Physicians, clinical officers, radiographers, and medical assistants (medical personnel) (*n* = 11) HIV-positive patients attending 3 different sites in Malawi (*n* = 183)	One public-private medical centre, one district hospital and one mission hospital	Malawi	To determine if remote expert radiology support would improve sonographer technique and interpretation in the FASH exam through the use of real-time quality feedback on image acquisition and interpretation.	Observational (prospective), quantitative, assessed effectiveness of the digital health intervention	Not provided	Not provided	The participant's interpretation of the ultrasound findings was compared to the expert radiologist/standard of reference (SOR)'s interpretation in 181 of the 183 exams. The clinicians identified 96 (6%) of these images as ‘abnormal’ while the SOR coded 85 (5%) as ‘abnormal’, revealing an overall agreement of 98% between the clinicians and the SOR. There was only one case of a false-positive effusion (i.e. where the participant erroneously identified a pericardial effusion as present while the SOR evaluated it as absent), resulting in an agreement rate of 99%.	Our prospective observational study suggests that both physician and non-physician clinicians can learn the technical skills of ultrasound image acquisition after a short four-day training course followed by focused tele-ultrasonography feedback.
24	Williamson, 2017	A reporting system to protect the human rights of people living with HIV and key populations	Key informants from government and civil society organisations (CSOs) (*n* = unspecified) People living with HIV and key populations (*n* = 50)	Community	Ghana	To outline findings from the implementation of a framework that protects the human rights of people living with HIV and key populations.	Mixed methods, quantitative, neither assessed efficacy nor effectiveness of the digital health intervention	Not provided	Not provided	Between December 1, 2013, and September 30, 2015, people living with HIV, key populations, and CSOs reported 50 cases of discrimination to Commission on Human Rights and Administrative Justice (CHRAJ). CSO-reported 28 (56%) of the cases through the (online) reporting system, 11 (22%) people reported in person, 10 (20%) people self-reported through reporting system and 1 (2%) person self-reported through SMS.	A reporting system can provide a critical link between people living with HIV, key populations, civil society, and national human rights institutions.
25	Willis, 2014	‘My story’-HIV positive adolescents tell their story through film	Adolescents attending support groups run by a non-governmental organisation (*n* = 12)	Community	Zimbabwe	To evaluate the digital storytelling process as a therapeutic approach to helping young people come to terms with the events in their lives and to develop coping strategies.	Qualitative, neither assessed efficacy nor effectiveness of the digital health intervention	Mean age was 20.75 years	7 (58.33%) were female	Storytellers found the process therapeutic as it helped them to move away from the negative themes which dominated their lives to a newer, richer perspective of their lives in which they had overcome challenges. Their films have provided caregivers and programmers with new insights into the challenges they faced and appropriate interventions for other adolescents living with HIV.	This digital storytelling process confirms to young people that their story really does matter. It validates their experiences and shows them that others place value in their experiences and the way they have overcome difficulties.

### Appraisal of included studies

Using the MMAT tool (see Supplementary Material 4), studies were grouped as qualitative (*n*  =  3),^47,51,52^ RCTs (*n*  =  3),^41,43,50^ quantitative non-randomised (*n*  =  8),^37,39,42,44,53–56^ quantitative descriptive (*n*  =  5)^35,36,38,48,49^ and mixed methods (*n*  =  6).^40,45,46,57–59^ Most studies met four to five criteria for their study design. Only three studies^36,41,56^ met three or fewer criteria.^36,41,56^

### Identification and description of the studies

#### WHO classification of digital health interventions

There were 13 studies^40–48,51, 53, 57, 58^ with digital health interventions targeted at clients as primary users and 12 studies^35–39,49,50,52,54–56,59^ with interventions targeted at healthcare providers as primary users. There were no digital health interventions targeted at health system managers or data services. For digital health interventions targeted at clients, targeted client communication^41,42,46,48^ and client-to-client communication^40,53,57,58^ were the most common ways clients accessed palliative care. Using the WHO classification, the digital health interventions used to provide palliative care to PLWH in SSA were categorised into 9 categories: targeted client communication,^41,42,46,48^ client to client communication,^40,53,57,58^ personal health tracking,^43,44,47^ citizen-based reporting,^45^ on-demand information services to clients,^51^ health care provider decision support,^50,56,59^ telemedicine,^35–39,49,54^ referral coordination, and laboratory and diagnostics imaging management.^55^ Across all approaches, telemedicine was the most commonly reported in the included studies^35–39,49,54^ (see Table 4).

**Table 4.** WHO classification and summarised TiDieR descriptions of digital health interventions used to deliver palliative care to PLWH in SSA.

WHO classification	Study	TIDieR description
Targeted client communication	^46^	Mobile phones were used to facilitate communication between healthcare providers and patients (including their treatment supporters). The communication included medication and clinic appointment reminders. This review was interested in the psychosocial support the PLWH received from their healthcare providers and treatment supporters. Software was used to automate sending of the SMSs to patients and treatment supporters. PLWH received the intervention in a community setting.
	^41^	Mobile phones were used to provide acceptance and commitment therapy to pregnant HIV-positive women to improve their psychological flexibility. Software automated the sending of SMSs to patients. The intervention included one face-to-face session within a clinic setting and was followed by weekly SMSs to mobile phones of the patients in their community.
	^42^	Mobile phones were used to provide remote counselling to adolescents living with HIV to improve their self-care capacity and adjustment to their illness. SMS, voice calls, multi-media and WhatsApp messages were used to administer the intervention. Healthcare workers made the voice calls and sent the messages. The adolescents received the intervention in their community.
	^48^	The study explored the acceptability of using mobile phones among PLWH to improve retention in care and adherence to treatment. This review was interested in the ‘retention in care’ component of the study because of its potential application to palliative care for PLWH. No intervention was administered to the participants.
Client to client communication	^40^	Social media support groups were used to provide HIV knowledge to adolescents living with HIV and to help the adolescents get social support, adhere to ART and stay in care. Online facilitators provided training, administered quizzes and moderated discussions. Participants were given data and feature phones to access the virtual groups. Initial face-to-face meetings were held by participants and their facilitators to agree on ground rules for the virtual support groups. The virtual support groups were used as a complementary to clinic visits.
	^57^	HIV-positive youth stable in care, provide social support to newly diagnosed HIV-positive youth through mobile phones. Phone calls, SMS and WhatsApp were used as communication channels. The virtual peer mentors guided newly diagnosed HIV-positive youth through the healthcare system. The mentors would conclude the mentorship process by inviting the mentees to a youth-adherence club.
	^58^	A chatroom of a social media platform was used to facilitate continued social support among HIV-positive youth who attended the same youth club. There was a moderator for the chatroom. Participants were given airtime to access the chatroom. The chatroom was complementary to clinic visits.
	^53^	A social media platform was used as a peer-support system to improve HIV/ART knowledge. Online moderators run a question and answer (Q&A) section of the platform. Participants used either their own electronic devices or computers installed in their clinics. The intervention was complementary to clinic visits.
Personal health tracking	^43^	Computer rehabilitation therapy was used as a palliative care intervention to improve cognitive skills for HIV-positive children. Multi-level brain training exercises were administered as part of a computer game that the children played. The intervention was administered face-to-face to individual children. Neuropsychologists decided which games the children played. A game with western focused content was used. The setting in which the children played the games was unclear.
	^44^	Computer rehabilitation therapy was used as a palliative care intervention to improve cognitive skills for HIV-positive children. Participants were controls from an earlier study.^43^ The intervention was multi-level brain training exercises administered as part of a computer game. The intervention was administered face-to-face to individual children. Trainers for the games were undergraduate psychologists/social workers. African content was used in the game. The intervention was administered in both clinic and community settings.
	^47^	Used digital storytelling as a psychosocial palliative care therapy for HIV-positive youth. The youth were mentored by a media expert and paediatric/adolescent HIV nurse counsellor to change negative narratives that dominated their lives to more positive narratives. The youth used computers to create their own stories and share with others. The study was conducted at an non-governmental organisation office that was familiar and safe for the children.
Citizen based reporting	^45^	PLWH, sex workers and MSM were provided with in-person, online and SMS interventions to report cases of discrimination when accessing healthcare, housing and other services to a government human rights body. The human rights body helped the complainants seek redress. This review was interested in the aspect of utilising digital technologies to tackle discrimination against PLWH as discrimination affects the psychological health of PLWH.^81^ Study participants utilised the online and SMS reporting in a community setting.
On demand information services to clients	^51^	The study^51^ explored some digital health interventions for provision of information to HIV-positive MSM in combination with other non-digital interventions. The study^51^ had both development and pilot phases. The development phase considered a mixture of digital and non-digital interventions including patient centred care, peer navigators, discrete pill carriers, pill taking/appointment reminders, information sharing on the internet and a telephone hotline. However, it appears that only patient centred care provided by healthcare workers, peer navigators (ART experienced MSM) and discrete pill carriers were tested in the pilot phase. The peer navigators provided guidance to ART naïve MSM using mobile phones and face-to-face. This review was interested in the potential to provide palliative care information and social support to HIV-positive MSM through the hotline and internet. The interest of the review also extended to use of mobile phones by ART experienced MSM to provide social support to ART naïve MSM to help them navigate HIV care. The study was conducted in both clinic and community settings.
Healthcare provider decision support	^59^	The intervention was software designed to standardise delivery of HIV counselling by health workers to PLWH. The intervention was administered face-to-face using a tablet. It was used for both individual and group counselling in a clinic setting.
	^56^	An application was used to diagnose neurocognitive impairment in HIV-positive patients. The intervention was delivered by lay health workers who were trained how to administer the app through a tablet. The app was administered to patients face-to-face in a clinic setting.
	^50^	The intervention was software designed to standardise delivery of HIV counselling by health workers to PLWH. It was administered through a computer face-to-face in a clinic setting. The intervention was used for individual counselling.
Telemedicine	^37^	The intervention was intended at diagnosing skin conditions in PLWHIV. A nurse who came face-to-face with patients received a few days training on how to use a dermatology application on a phone. The nurse captured and forwarded skin and oral images of patients using a mobile phone to a secure password protected site. Internet was used to support this store and forward technique. Remote dermatology experts reviewed the images. The diagnoses and treatment recommendations of the remote experts were compared to an onsite US board certified dermatologist whose diagnoses and treatment recommendations were used as the gold standard. The intervention was delivered in a clinic/hospital setting.
	^38^	This is another report on the above study.^37^
	^35^	Tele-sonography was used to diagnose HIV-associated extrapulmonary tuberculosis (TB). A local physician with one-week training in abdominal ultrasound had face-to-face consultations with patients. The physician was supported by a remote expert in live online interactions during patient examination. The examinations were done in a clinic,
	^36^	The intervention was used to diagnose and recommend treatment for neurological symptoms of different diseases. This review focused on the use of the intervention in PLHIV. Local healthcare workers who interacted face-to-face with the patients received 3-4 weeks training. The local healthcare workers forwarded patient history, examination and their questions to a remote neurologist using internet through a web platform. The intervention was provided in health centres.
	^39^	Remote cervical cancer screening was provided to HIV-positive women. Medical students took in-person photos of the cervixes of the women with a mobile phone camera. The students underwent a day's training in taking the pictures. The photos were transmitted by multi-media messaging (MMS) and stored in a database for evaluation by nurse midwives. The photos were also shared with a remote expert gynaecologist for evaluation. The use of MMS eliminated the need for internet at the clinic where the intervention was provided.
	^49^	Evaluators carried out face-to-face neurocognitive development testing in HIV-affected children. The evaluators had college education and underwent one-week basic neurodevelopment assessment training. They videotape themselves while assessing each child and uploaded videos online where quality assurance centre staff remotely access the files for review. There was also an onsite supervisor that provided support to the evaluators in addition to the remote experts. The setting in which the intervention was provided is unclear.
	^54^	Tele-sonography was used to diagnose HIV-associated extrapulmonary TB. Local health workers that came face-to-face with the patients had a 4-day training on focused assessment with sonography for HIV (FASH). The healthcare workers captured and sent images to a remote United States board-certified radiologist with expertise in ultrasonography. The intervention was administered in a hospital setting.
Referral coordination	^52^	The study^52^ explored the need for an mHealth intervention to help coordinate linkage to clinics for HIV-positive patients found through a home-based HIV testing program delivered by community health workers (CHWs). No intervention was tested. This review was interested in the potential of the intervention to link PLWH to counselling and other palliative care services among a host of HIV care services provided at HIV clinics.
Laboratory and diagnostic imaging management	^55^	The intervention diagnosed atrial fibrillation (AF) in HIV-positive patients to prevent complications of untreated atrial fibrillation such as ischemic stroke. The goal was to diagnose and treat the physical symptom to improve the quality of life of PLWH. Trained medical students administered the intervention to individual patients face-to-face in a clinic. A patient's electrocardiogram (EGC) recording was transferred from the ECG device to a smartphone for interpretation.

#### Description of digital health interventions aligned with the TIDieR checklist

Summarised TIDieR descriptions of the digital health interventions are provided alongside alignment with WHO classification in Table 4. Detailed descriptions are provided in Supplementary Material 3.

#### Stage of digital health intervention development

**Figure 2.**
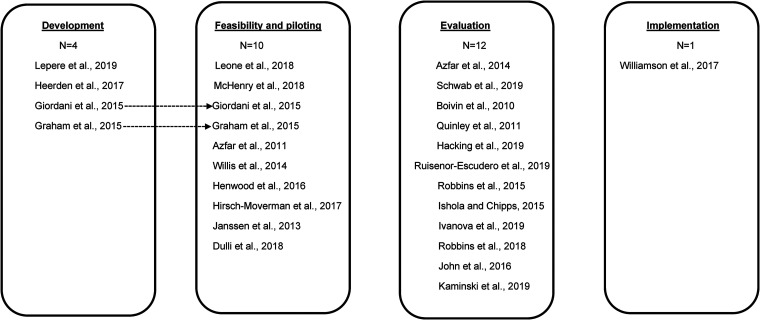
Stages of digital health interventions.

There were four studies^44,48,51,52^ at the development stage. The second largest number of studies (*n*  =  10)^35,36,38,40,44,46,47,51,58,59^ were at the feasibility and piloting stage. The largest number of studies (*n*  =  12)^37,39,41–43,49,50,53–57^ were at the evaluation stage. Only one study^45^ reported research at the implementation stage (see Figure 2).

#### Reported efficacy and effectiveness of digital health interventions

Both efficacy and effectiveness of digital health interventions are reported in 13 of the 25 included studies.^37,39,41–44,49,50,53–57^

A full RCT^41^ assessed the efficacy and a pre-post-test study^42^ assessed the effectiveness of targeted client communication: (a) delivery of acceptance and commitment therapy through SMS was efficacious in improving psychological flexibility of pregnant HIV-positive women,^41^ and (b) mobile phone counselling was also effective in improving psychological outcomes of undisclosed HIV positive youth.^42^

A mixed methods and pre-post-test study evaluated the effectiveness of client-to-client communication^53,57^: (a) providing psychosocial support to HIV-positive youth through mobile phones was effective in getting them to commence ART and complete viral load tests,^57^ and (b) peer psychosocial support through a social media platform was effective in improving adherence intentions for HIV positive youth.^53^

A pilot RCT^43^ assessed the efficacy and a pre-post-test study^44^ assessed the effectiveness of personal health tracking interventions.^43,44^ Computer rehabilitation therapy was efficacious in improving the neurocognitive skills of HIV-positive children.^43^ Computer rehabilitation therapy was also effective in improving neurocognitive skills of HIV-positive children.^44^

The effectiveness of healthcare provider decision support interventions was assessed in a cross-sectional study,^56^ and the efficacy of healthcare decision support interventions was also assessed in a pilot RCT^50^: (a) a mobile phone application was effective in diagnosing neurocognitive impairment in HIV-positive patients,^56^ and (b) HIV counselling through a digital platform was efficacious in improving psychosocial outcomes of HIV positive adults.^50^

Two cross-sectional studies^37,39^ and two observational studies^49,54^ assessed the effectiveness of telemedicine interventions: (a) teledermatology was not effective in diagnosing and recommending treatment for skin conditions of PLWH,^37^ (b) remote diagnosis of cervical cancer in HIV-positive women was effective,^39^ (c) teleultrasonography was effective in supervising medical personnel that diagnosed HIV associated tuberculosis^54^ and (d) a remote quality assurance centre was effective in helping neurodevelopment evaluators maintain the quality of their testing.^49^

A prospective cross-sectional study assessed the effectiveness of a laboratory and diagnostics imaging management intervention.^55^ A portable electrocardiogram (ECG) device was effective in generating readable ECGs in patients with the World Health Organisation AIDS Clinical Staging (WACS) of 1 only.^55^ Table 5 details included studies outlining the efficacy and effectiveness of digital health interventions for provision of palliative care in PLWH in SSA.

**Table 5.** Efficacy and effectiveness of digital health interventions for provision of palliative care in PLWH in SSA.

Category of intervention	Author (year)/country	Aim/design/theoretical model	Sample	Interventions	Results
Targeted client communication	Ishola (2015), Nigeria	To develop, implement and evaluate acceptance and commitment therapy (ACT) in prevention of mother to child HIV transmission (PMTCT) programmes in Nigeria using weekly mobile phone messages with the aim of increasing psychological flexibility of HIV-positive pregnant women in Nigeria. RCT. Relational frame theory. Evaluated efficacy of the digital health intervention.	*N* = 132, mean age 31.6 years. *n* = 33, intervention group 1. Mean age 31.9 (SD = 4.42) years *n* = 33 intervention group 3. Mean 32.1 (SD = 4.5) years *n* = 33 control group 2. Mean 31.3 (SD = 4.5) years *n* = 33 control group 4. Mean 31.4 (SD = 4.6) years	The intervention groups received post HIV test counselling and were exposed to three sessions of ACT followed by weekly value-based health messages. The control groups only received the post-HIV test counselling (Standard of care).	A significant increase in psychological flexibility was observed in pregnant HIV-positive women post-test in the intervention group (*t* = 3.4, *p* < .001). However, some pre-test sensitisation was suspected because of the significant interaction that was found between the intervention and pre-test factors after analysis of variance (*F*(1,33) = 19.2, *p* < .001).
	John (2016), Nigeria	Evaluating the effectiveness of mobile phones in enhancing self-care, adjustment and engagement in non-disclosed youth living with HIV in Nigeria. Pre- and post-test. Evaluated effectiveness of the digital health intervention.	*N* = Age 19. range 15–29 years	Participants received HIV information, motivation and counselling through mobile phones over a three months duration. The comparator was no intervention.	There was an improvement in the mean self-care capacity scores for the participants from 21.6 (baseline) to 45.8 (*p* < .001) and 51.5 (*p* = .02) at 3 months and 6 months post-intervention respectively. Scores for psychological adjustment, mainly in keeping a sense of self-worth, also improved from 25.6% to 58.9% (*p* < .001) at 3 months and 103.3 (*p* < .001) at 6 months post-test. Engagement scores increased from 43.4 (passivity stage) to 56.1(stage of action) and 65.8 (stage of action) at 3 months and 6 months post-test, respectively. No significant change occurred in utilisation of formal HIV healthcare services as 78.9% of the participants still chose to consult healthcare workers using mobile phones.
Client to client communication	Hacking (2019), South Africa	To determine if peer-to-peer mentorship, specifically between newly diagnosed HIV-positive youths and HIV-positive youths stable in care, could be successfully implemented using mobile phones as the primary means of communication. Mixed methods (matched case-control and in-depth qualitative interviews). Evaluated effectiveness of the digital health intervention.	*N* = 105 *n* = 35 intervention. Median 20 years, 5 months. *n* = 70 control. Median 22 years, 7 months.	Virtual mentor would interact with the mentee via a mobile interface (SMS text messaging, call or WhatsApp messenger). The control group received no intervention.	Higher rates of ART initiation 28/35 (80%) in intervention group compared to matched controls 30/70 (42%). Higher rates of viral load completion 28/35 (80%) compared to controls 32/70 (45%). No differences in viral suppression and retention in care at both 6 and 12 months.
	Ivanova (2019), Kenya	To evaluate the usability and the effectiveness of a pilot digital peer support system in improving HIV/ART knowledge, perceived importance of adherence, perceived self-efficacy in adhering and future intentions towards adherence. Pre- and post-test. Evaluated effectiveness of the digital health intervention.	*N* = 90. Mean age 18.4 years	Project coordinators, health care providers and young people wrote blog posts on different topics related to sexual and reproductive health, HIV, medication, nutrition, relationships, etc. and shared them on the social media platform. Healthcare providers run Q&A section. The comparator was no intervention.	Knowledge scores for participants improved by 0.3 points, Wilcoxon signed ranks test showed no statistical significance (-0.26). No significant difference pre- and post-intervention for perceived importance in maintaining adherence (*p* = .84), self-efficacy/confidence (*p* = .31) and missed doses (*p* = .95). Wilcoxon signed ranks test showed significant difference for pre- and post-intervention adherence intentions (-0.03).
Personal health tracking	Boivin (2010), Uganda	To establish the feasibility and gather preliminary evidence for the effectiveness of computerised cognitive rehabilitation therapy with African children in a low-resource setting, as a proof-of-concept for the potential of such an intervention for HIV-affected children globally. RCT. Evaluated efficacy of the digital health intervention.	*N* = 60 *n* = 32 intervention. Mean 10.34 years *n* = 28 control. Mean 9.36 years	Children in the intervention arm were given appointments to return for twice weekly game sessions over 5 weeks. Children in the control arm did not receive any cognitive intervention.	Significant improvements on card detection speed (mean difference = 0.07, SE = 0.02, *p* = .01) and maze learning (mean difference = -0.07, SE = 0.02, *p* = .001) in intervention group. Similar results after adjusting for age, school grade level, standardised weight for age, baseline performance on the Kaufman Assessment Battery for Children, 2^nd^ edition (KABC-II), socioeconomic status and gender in an analysis for covariance: card detection speed (group effect = 0.06, SE = 0.02, *p* = .02) and maze learning (group effect = -0.06, SE = 0.02, *p* < .001).
	Giordani (2015), Uganda	To develop a computer-based training platform, BPG, suitable for use with children within a rural, sub-Saharan Africa setting and then complete an initial field trial with that program. Pre- and post-test Evaluated effectiveness of the digital health intervention.	*N* = 33. Mean 8.55 years.	Participants in this study were controls in Boivin et al.’s (2010) study where they were exposed to no intervention. In this study they were trained to play Brain Powered Games (BPG). The comparator was no intervention.	Clinically significant improvements with large effect sizes for correct moves per second in the CogState Groton Maze Learning Test Learning Score (effect size = 1.09, *p* < .01) and CogState Groton Maze Learning Test Chase Score (effect size = 1.29, *p* < .01). A large effect size for test of variables of attention (TOVA) response time in milliseconds (effect size = 0.79, *p* < .01). A mild effect size for TOVA percent omission errors (effect size = 0.53, *p* < .01).
Healthcare provider decision support	Robbins (2015), South Africa	To examine medication adherence and key psychosocial outcomes among non-adherent South African HIV-positive patients, on antiretroviral therapy (ART) who were randomised to receive either Masivukeni (a digital health intervention) or standard of care (SOC) counselling for ART non-adherence. RCT. Evaluated efficacy of the digital health intervention.	*N* = 65 *n* = 33 intervention. Mean 38.4 years. *n* = 32 control. Mean 38.4 years.	In the first session the counsellor used a computer programme to assess PLWH's psychiatric distress and alcohol/substance abuse. The computer programme was also used to provide 5 subsequent sessions of HIV medication counselling to the patient and a support partner. The control group was exposed to standard of care counselling by other counsellors which was often a single session of less than 15 min.	Intervention participants expressed more positive attitudes with regards to disclosure of their HIV status (*p* = .04). More self-reported medication social support to take ARVs in intervention group (*p* = .02). Self-reported decrease in social rejection due to HIV (*p* = .02) and improved clinic–patient relationship by close to 5 points in intervention group (*p* = .05).
	Robbins (2018), South Africa	To evaluate the ability of the lay health worker-administered NeuroScreen app to detect neurocognitive impairment in PLWH, as defined by a gold standard neuropsychological test battery. Cross-sectional. Evaluated effectiveness of the digital health intervention.	*N* = 102. Mean 33.31 years.	Trained lay health workers administered NeuroScreen to PLWH. The gold standard comparator was a neuropsychology battery test administered by neuropsychology technicians using pencil and paper (standard of care).	The NeuroScreen Total Score 1 (Sum of all tests) had a sensitivity of 81.48% (95% CI: 61.92–93.70%) and specificity of 74.67% (95% CI: 63.30–84.01%). While the NeuroScreen Total Score 2 (sum of all tests and available error scores) provided a sensitivity of 81.48% (95% CI: 61.92–93.70%) and specificity of 81.33% (95% CI: 70.67–89.40%). The NeuroScreen Total Score 3 (sum of four tests) had sensitivity of 92.59% (95% CI: 75.71–99.09%) and specificity of 70.67% (95% CI: 59.02–80.62%).
Telemedicine	Azfar (2014), Botswana	To determine whether the use of mobile tele-dermatology technology in HIV-positive patients in Gaborone, Botswana, was reliable and produced valid assessments compared with face-to-face dermatologic consultations. Cross-sectional. Evaluated effectiveness of the digital health intervention.	*N* = 76. Median age 39 years	Patient dermatology images were forwarded from a Samsung Soul SGH-U900 cellular phone with a 5-megapixel camera to a secure password protected teledermatology evaluation website. Remote evaluations of the skin conditions were completed by 3 US-based board-certified dermatologists and 1 board-certified oral medicine specialist The gold standard comparator was diagnosis and treatment recommendations of a face-to-face US board certified dermatologist (standard of care).	The agreement on primary diagnosis ranged from 47% (K: 0.41; 95% CI: 0.31–0.52) to 57% (K: 0.51; 95% CI: 0.41–0.61). Agreement on treatment of the primary diagnosis ranged from 32% (K: 0.08; 95% CI: 0.02 to 0.15) to 51% (K: 0.12; 95% CI: 0.01–0.23). In a subset of cases with oral lesions, where the face-to-face dermatologist was compared to an oral medicine and dentistry expert, the interrater agreement ranged from 62–68% (K: 0.51–0.58) for the primary diagnosis. The K coefficient for treatment ranged from −0.14 to 0.09.
	Quinley (2011), Bostwana	To determine whether mobile telemedicine is safe and effective for cervical cancer screening when employed as an adjunct to visual inspection with acetic acid (VIA). Cross-sectional. Evaluated effectiveness of the digital health intervention.	*N* = 99. Median 34 years	Diagnosis of cervical cancer by nurses and a remote expert using images of women's cervixes. The gold standard comparator was face-to-face diagnosis of cervical cancer in women using visual inspection with acetic acid (standard of care). This was done by the nurses.	The nurses agreed with their previous face-to-face diagnosis in 70% of the cases (K: 0.38, *p* < .001). When compared to a remote expert gynaecologist, there was 89% and 82% agreement for negative and positive results, respectively (K: 0.71, *p* < .001).
Telemedicine	Ruisenor-Escudero (2019)	To describe and discuss how a quality assurance (QA) model for neurodevelopment testing can be used across settings and with personnel of varying experience and backgrounds. Observational. Evaluated effectiveness of the digital health intervention.	*N* = 615. Mean age 6.6–8.0 years across 4 countries	A remote quality assurance (QA) centre was used to mentor newly trained neurodevelopment testing evaluators, and evaluator performance in administering the tests was assessed by remote neurodevelopment testing experts. Neurodevelopment evaluators uploaded videos of themselves evaluating children into a previously set up file hosting service (e.g. Dropbox) where the QA centre staff could remotely access files for internal review. There was no comparator to the intervention.	The average total score of the evaluators at baseline was 161 (range: 118–180), after 10 months of the QA centre's supervision the average total score was 178 (range: 175–180), and the average total score for the last video evaluators had submitted to the QA centre was 165 (range: 140–180). The authors considered these rubric scores sufficient because a mean score greater than 70% of the maximum score is what was required.
	Schwab (2019), Malawi	To determine if remote expert radiology support would improve sonographer technique and interpretation in the FASH exam through the use of realtime quality feedback on image acquisition and interpretation. Observational. Evaluated effectiveness of the digital health intervention.	*N* = 183. Age of participants was not provided.	Diagnoses of a remote expert radiologist were used as a reference against 11 newly trained clinicians who performed focused assessment with sonography for HIV (FASH). The gold standard was diagnoses of the remote expert radiologist (not standard of care).	The clinicians classified 96 (6%) of the sonography images as abnormal while the radiologist classified 85 (5%) as abnormal; thus, an overall agreement of 98%. There was a 99% agreement between the radiologist and the clinicians in identification of pericardial effusion, that is, only one false-positive from one clinician. The agreement in diagnosing peri-portal and para-aortic lymphadenopathy was 98%, the clinicians missed 2 cases of peri-portal and 1 case of para-aortic lymphadenopathy. The radiologist and clinicians had an agreement of 99% in identifying ascites, splenic lesions and liver lesions. There were 14 other abnormalities identified by clinicians, while the radiologist only found 9, resulting in an agreement rate of 94%.
Laboratory and diagnostics imaging management	Kaminski (2019), Kenya	To investigate the quality of the ECG signal acquired by a touch ECG device (Kardia) in patients with different clinical stages of established HIV infection. Cross-sectional study. Evaluated effectiveness of the digital health intervention.	*N* = 263. Median age 46 (39–53) years.	A portable ECG was used as an intervention to detect atrial fibrillation in PLWH. There was no comparator for the intervention.	In comparison to patients with World Health Organisation AIDS Clinical Staging (WACS) equal to 1, patients with a WACS greater than 1 had a four-fold higher risk of having an unreadable electrocardiogram (ECG) using a Kardia device, unadjusted (OR: 4.25; 95% CI: 2.33–7.73; *p* < .0001). The risk was very similar when the analysis was adjusted for participants age, body mass index and duration from HIV diagnosis (OR: 3.95; 95% CI: 2.14–7.29; *p* < .0001).

## Discussion

There is an emerging evidence base of good quality research to inform digital health interventions for the provision of palliative care to PLWH in SSA. In SSA, digital health has been used to provide palliative care to PLWH through targeted client communication, client-to-client communication, personal health tracking, citizen-based reporting, on-demand information services to clients, healthcare provider decision support, telemedicine, referral coordination and laboratory and diagnostics imaging management. Most interventions were at the stages of feasibility, piloting and evaluation. Of the included studies, efficacy was reported across three studies and effectiveness across 10 studies for digital health interventions seeking to improve intended outcomes. Whilst previous research has focused on SMS interventions,^60^ there are now multiple, broader digital health approaches being developed and tested to support patients with HIV and palliative care needs. However, further evidence is needed to understand how the effectiveness of interventions is realised and how they can best be integrated into the routine delivery of palliative care services.

Telemedicine was the most researched digital health intervention for palliative care in PLWH. A human resource gap in SSA, particularly at the primary care level, has been highlighted^61^ with telemedicine posing a potential approach to addressing the shortage of expertise. However, the use of telemedicine was limited to provider-to-provider communication in a clinical setting making it mostly applicable to the district hospital and specialist palliative care models. In SSA, there is scope to explore telemedicine in the provision of palliative care for PLWH by extending its use to facilitate provider-client communication. In developed countries, telemedicine has been used to remotely manage pain and other symptoms, expanding the reach of palliative care services.^62^ It has also been used to coordinate patient care, minimising utilisation of outpatient services^63^ while improving the provider–client relationship.^64^ Such an approach, encouraging telehealth programs to enable, for example, families to virtually visit and partake in health decisions with loved ones, has been a recommendation of the World Health and Palliative Care Alliance in response to the COVID-19 pandemic.^65^ Critical to expansion to approaches is the need to accommodate low digital health literacy for both providers and patients, alongside other known barriers including the cost of delivering telemedicine and telecommunication and infrastructure challenges (e.g. intermittent electrical supply, limited mobile phone network coverage), especially in rural areas.^66^ Additional, research is needed in SSA to determine the cost, available infrastructure for digital health, and acceptability of telemedicine among patients and providers.

The second most researched digital health interventions for palliative care were targeted client communication and client-to-client communication. The underlying theme across both categories was the use of digital health to provide psychosocial support to PLWH, with a great need for psychosocial support reported previously among PLWH.^67^ Studies conducted in several SSA countries found the prevalence of mental illness among PLWH to be 19% or higher.^68^ This review found that the use of digital health psychosocial interventions among PLWH occurred in both community and clinical settings, suggesting their potential appropriateness across different models of palliative care in SSA (i.e. community, district and specialist).^69^ Digital health interventions that provide counselling and peer support may be an approach to supporting psychosocial symptoms among PLWH in SSA, with a number of the interventions having demonstrated acceptability and effectiveness in assessing, monitoring and treating severe mental illnesses in LMICs.^70^

The literature on the use of digital health to support palliative care in SSA is at an early stage and there remains a need to understand the needs and preferences of PLWH and the services delivering palliative care to inform the requirements of digital health interventions. A review^71^ of successful approaches for scaling up digital health interventions in LMICs found that interventions that align with need are more likely to be adopted and engaging for end-users. In addition, understanding user needs helps reduce health inequalities by ensuring the engagement of vulnerable groups from the outset.^72^ Alongside determining user needs, future research exploring digital health for PLWH in SSA needs to explore the mechanisms that underpin and mediate any changes to outcomes arising from interventions. Exploring such mechanisms in well-established digital health approaches (e.g. telemedicine) may offer intervention agnostic insights that could inform the development of less common approaches that have value for PLWH in SSA. Such findings may also have relevance beyond the SSA region as the lack of an evidence base and end-user involvement in digital health interventions for PLWH is an issue across many LMICs.^73^ Within this review, only one study^41^ developed a digital health intervention underpinned by an existing theory. Exploring opportunities for applying and developing underlying theories that can inform digital health intervention development is a crucial next step in developing the research field.^74^ Furthermore, exploring cost-effectiveness evaluation of digital health interventions that have demonstrated effectiveness in improving patient outcomes is necessary to facilitate scale-up and wider adoption, with cost a central consideration for government decision-making around intervention adoption.^75^

## Strengths and limitations

To our knowledge, this is the first systematic review to explore the role of digital health in palliative care for PLWH in SSA. This review utilised robust search strategies with broad inclusion criteria, including any study design. This review is, however, limited by the fact that no searches were undertaken in grey literature databases and that hand-searching was not done due to time limitations. As a result, while we are confident of the inclusion of a wide body of literature, it may not reflect the entirety of relevant research literature.

## Conclusion

Research into the use of digital health interventions to support palliative care for people living with HIV in SSA is developing. However, there is a lack of a theoretical underpinning to many interventions, the mechanisms through which interventions lead to change in patient outcomes are not clear, and most reported interventions have not progressed to a stage of implementation as part of routine care. Future research should focus on embedding theory into intervention development for PLWH, exploring the potential of additional digital health interventions beyond primarily telemedicine approaches, and aligning intervention development with the wider regional need for the expansion of palliative care provision.

## Supplemental Material

sj-xlsx-1-dhj-10.1177_20552076221133707 - Supplemental material for The role of digital health in palliative care for people living with HIV in sub-Saharan Africa: A systematic review Click here for additional data file. Supplemental material, sj-xlsx-1-dhj-10.1177_20552076221133707 for The role of digital health in palliative care for people living with HIV in sub-Saharan Africa: A systematic review by Christopher Mwase, Kennedy Nkhoma and Mathew J Allsop in Digital Health

sj-docx-2-dhj-10.1177_20552076221133707 - Supplemental material for The role of digital health in palliative care for people living with HIV in sub-Saharan Africa: A systematic review Click here for additional data file. Supplemental material, sj-docx-2-dhj-10.1177_20552076221133707 for The role of digital health in palliative care for people living with HIV in sub-Saharan Africa: A systematic review by Christopher Mwase, Kennedy Nkhoma and Mathew J Allsop in Digital Health
