# Supplementary material for: The role of digital health in palliative care for people living with HIV in sub-Saharan Africa: A systematic review
Source: Digit Health. 2022 Nov 22;8:20552076221133707. doi: 10.1177/20552076221133707 (PMC9706081; doi:10.1177/20552076221133707)
Supplement: sj-docx-2-dhj-10.1177_20552076221133707 - Supplemental material for The role of digital health in palliative care for people living with HIV in sub-Saharan Africa: A systematic review [file sj-docx-2-dhj-10.1177_20552076221133707.docx]

# Supplementary Materials

## **Supplementary Materials 1**: Search strategies for MEDLINE, Embase, PyschINFO and Global Health

**Database(s): Ovid MEDLINE(R) and Epub Ahead of Print, In-Process & Other Non-Indexed Citations, Daily and Versions(R) 1946 to May 21, 2020
Search Strategy:**

| **#** | **Searches** | **Results** |
| --- | --- | --- |
| 1 | exp HIV-2/ or exp HIV/ or exp HIV-1/ | 98189 |
| 2 | exp Acquired Immunodeficiency Syndrome/ | 76187 |
| 3 | exp hiv/ or exp hiv infections/ | 313950 |
| 4 | (human immunodeficiency or human immunodeficiency virus or human immuno-deficiency virus or human immune-deficiency virus).tw. | 87552 |
| 5 | (hiv or hiv-1* or hiv-2* or hiv1 or hiv2 or human immunodeficiency virus or human immunedeficiency virus or human immuno-deficiency virus or human immune-deficiency virus or (human immun* and deficiency virus) or acquired immunedeficiency syndrome or acquired immuno-deficiency syndrome or acquired immune-deficiency syndrome or (acquired immun* and deficiency syndrome)).tw. | 330815 |
| 6 | HIV.tw. | 309147 |
| 7 | (Immun* adj3 deficienc* adj3 virus*).tw. | 1235 |
| 8 | Acquired Immunodeficienc*.tw. | 16632 |
| 9 | exp Electronic Health Records/ | 19767 |
| 10 | exp eHealth/ | 27943 |
| 11 | exp mHealth/ | 27943 |
| 12 | exp Internet/ | 78396 |
| 13 | exp Microcomputers/ | 21905 |
| 14 | exp Therapy, Computer-Assisted/ | 63530 |
| 15 | exp Cell Phones/ | 10528 |
| 16 | exp Smartphone/ | 4215 |
| 17 | exp MP3-Player/ | 183 |
| 18 | exp Computers, Handheld/ | 7683 |
| 19 | exp Telemedicine/ | 27943 |
| 20 | exp Videoconferencing/ or Webcasts/ | 2692 |
| 21 | exp Text Messaging/ | 2801 |
| 22 | exp Telenursing/ | 215 |
| 23 | exp Mobile Applications/ | 5679 |
| 24 | exp Social Media/ | 7522 |
| 25 | exp Reminder Systems/ | 3431 |
| 26 | exp Electronic Mail/ | 2667 |
| 27 | exp Multimedia/ | 1917 |
| 28 | exp Hypermedia/ | 398 |
| 29 | exp Blogging/ | 974 |
| 30 | Game.tw. | 24509 |
| 31 | ((cell* or mobile*) adj3 (phone* or telephone* or technolog* or device*)).tw. | 28740 |
| 32 | (smartphone* or smart-phone* or cellphone* or mobiles).tw. | 11932 |
| 33 | ((personal adj3 digital) or (PDA adj3 (device* or assistant*)) or MP3 player* or MP4 player*).tw. | 1444 |
| 34 | (samsung or nokia).tw. | 1170 |
| 35 | (windows adj3 (mobile* or phone*)).tw. | 51 |
| 36 | Android.tw. | 2460 |
| 37 | (ipad* or i-pad* or ipod* or i-pod* or iphone* or i-phone*).tw. | 2680 |
| 38 | (tablet* adj3 (device* or computer*)).tw. | 1584 |
| 39 | (mhealth or m-health or "mobile health" or ehealth or e-health or "electronic health").tw. | 24423 |
| 40 | (telemedicine or tele-medicine or telehealth or tele-health or telecare or tele-care or telenursing or tele-nursing or telepsychiatry or tele-psychiatry or telemonitor* or tele-monitor* or teleconsult* or tele-consult* or telecounsel* or tele-counsel* or telecoach* or telecoach*).tw. | 16318 |
| 41 | (videoconferenc* or video-conferenc* or webcast* or web-cast*).tw. | 2995 |
| 42 | (((text* or short or voice or multimedia or multi-media or electronic or instant) adj3 messag*) or instant messenger).tw. | 5823 |
| 43 | (texting or texted or texter* or ((sms or mms) adj (service* or messag*)) or interactive voice response* or IVR or voice call* or callback* or voice over internet or VOIP).tw. | 3356 |
| 44 | (Facebook or Twitter or Whatsapp* or Skyp* or YouTube or "You Tube" or Google Hangout*).tw. | 7777 |
| 45 | "mobile app*".tw. | 3866 |
| 46 | (social adj3 (media or network*)).tw. | 28971 |
| 47 | (remind* adj3 (text* or system* or messag*)).tw. | 1807 |
| 48 | (electronic mail* or email* or e-mail or webmail).tw. | 15322 |
| 49 | (e-BI or e-SBI or ehealth or e-health or electronic health or mhealth or m-health or mobile health or virtual health or digital health or technological aid?).tw. | 25954 |
| 50 | ((blackberr* or black-berr*) adj3 (mobile* or phone* or computer*)).tw. | 21 |
| 51 | (google adj3 phone*).tw. | 9 |
| 52 | exp Africa South of the Sahara/ | 206762 |
| 53 | "Sub-Saharan Africa*".tw. | 22731 |
| 54 | African*.tw. | 144085 |
| 55 | (Angola* or Benin* or Botswana* or "Burkina Faso" or Burundi* or Cameroon* or "Cape Verde*" or "Cabo Verde*" or Chad or Comoros or Congo* or "Ivory Coast" or "Cote d'Ivoire" or Djibouti* or Equatorial Guinea or Eritrea* or Ethiopia* or Gabon* or Gambia* or Guinea* or Kenya* or Lesotho or Liberia* or Madagasca* or Malawi* or Mali or Mauritania* or Mauriti* or Mozambi* or Namib* or Niger* or Nigeria* or Rwanda* or "Sao Tome*" or Principe* or Senegal* or Seychelles or "Sierra Leone" or Somali* or South Africa or Sudan* or Swazi* or Eswantini* or Tanzania* or Togo* or Uganda* or Western Sahara or Zambia* or Zimbabw*).tw. | 336538 |
| 56 | 1 or 2 or 3 or 4 or 5 or 6 or 7 or 8 | 403452 |
| 57 | 9 or 10 or 11 or 12 or 13 or 14 or 15 or 16 or 17 or 18 or 19 or 20 or 21 or 22 or 23 or 24 or 25 or 26 or 27 or 28 or 29 or 30 or 31 or 32 or 33 or 34 or 35 or 36 or 37 or 38 or 39 or 40 or 41 or 42 or 43 or 44 or 45 or 46 or 47 or 48 or 49 or 50 or 51 | 326969 |
| 58 | 52 or 53 or 54 or 55 | 504945 |
| 59 | 56 and 57 and 58 | 1196 |

**Database(s): Embase Classic+Embase 1947 to 2020 May 21
Search Strategy:**

| **#** | **Searches** | **Results** |
| --- | --- | --- |
| 1 | exp HIV-2/ or exp HIV/ or exp HIV-1/ | 194003 |
| 2 | exp Acquired Immunodeficiency Syndrome/ | 140242 |
| 3 | exp hiv/ or exp hiv infections/ | 477745 |
| 4 | (human immunodeficiency or human immunodeficiency virus or human immuno-deficiency virus or human immune-deficiency virus).tw. | 95600 |
| 5 | (hiv or hiv-1* or hiv-2* or hiv1 or hiv2 or human immunodeficiency virus or human immunedeficiency virus or human immuno-deficiency virus or human immune-deficiency virus or (human immun* and deficiency virus) or acquired immunedeficiency syndrome or acquired immuno-deficiency syndrome or acquired immune-deficiency syndrome or (acquired immun* and deficiency syndrome)).tw. | 415928 |
| 6 | HIV.tw. | 391867 |
| 7 | (Immun* adj3 deficienc* adj3 virus*).tw. | 1461 |
| 8 | Acquired Immunodeficienc*.tw. | 17518 |
| 9 | exp Electronic Health Records/ | 17247 |
| 10 | exp eHealth/ | 44653 |
| 11 | exp mHealth/ | 0 |
| 12 | exp Internet/ | 109027 |
| 13 | exp Microcomputers/ | 14648 |
| 14 | exp Therapy, Computer-Assisted/ | 15156 |
| 15 | exp Cell Phones/ | 27869 |
| 16 | exp Smartphone/ | 11892 |
| 17 | exp MP3-Player/ | 200 |
| 18 | exp Computers, Handheld/ | 1437 |
| 19 | exp Telemedicine/ | 39206 |
| 20 | exp Videoconferencing/ or Webcasts/ | 4097 |
| 21 | exp Text Messaging/ | 4815 |
| 22 | exp Telenursing/ | 270 |
| 23 | exp Mobile Applications/ | 11292 |
| 24 | exp Social Media/ | 19430 |
| 25 | exp Reminder Systems/ | 2596 |
| 26 | exp Electronic Mail/ | 21854 |
| 27 | exp Multimedia/ | 3989 |
| 28 | exp Hypermedia/ | 387 |
| 29 | exp Blogging/ | 327 |
| 30 | Game.tw. | 28400 |
| 31 | ((cell* or mobile*) adj3 (phone* or telephone* or technolog* or device*)).tw. | 37523 |
| 32 | (smartphone* or smart-phone* or cellphone* or mobiles).tw. | 16853 |
| 33 | ((personal adj3 digital) or (PDA adj3 (device* or assistant*)) or MP3 player* or MP4 player*).tw. | 1940 |
| 34 | (samsung or nokia).tw. | 2283 |
| 35 | (windows adj3 (mobile* or phone*)).tw. | 83 |
| 36 | Android.tw. | 4058 |
| 37 | (ipad* or i-pad* or ipod* or i-pod* or iphone* or i-phone*).tw. | 5208 |
| 38 | (tablet* adj3 (device* or computer*)).tw. | 2505 |
| 39 | (mhealth or m-health or "mobile health" or ehealth or e-health or "electronic health").tw. | 32650 |
| 40 | (telemedicine or tele-medicine or telehealth or tele-health or telecare or tele-care or telenursing or tele-nursing or telepsychiatry or tele-psychiatry or telemonitor* or tele-monitor* or teleconsult* or tele-consult* or telecounsel* or tele-counsel* or telecoach* or telecoach*).tw. | 22141 |
| 41 | (videoconferenc* or video-conferenc* or webcast* or web-cast*).tw. | 4431 |
| 42 | (((text* or short or voice or multimedia or multi-media or electronic or instant) adj3 messag*) or instant messenger).tw. | 7590 |
| 43 | (texting or texted or texter* or ((sms or mms) adj (service* or messag*)) or interactive voice response* or IVR or voice call* or callback* or voice over internet or VOIP).tw. | 4835 |
| 44 | (Facebook or Twitter or Whatsapp* or Skyp* or YouTube or "You Tube" or Google Hangout*).tw. | 11330 |
| 45 | "mobile app*".tw. | 5183 |
| 46 | (social adj3 (media or network*)).tw. | 36529 |
| 47 | (remind* adj3 (text* or system* or messag*)).tw. | 2533 |
| 48 | (electronic mail* or email* or e-mail or webmail).tw. | 31928 |
| 49 | (e-BI or e-SBI or ehealth or e-health or electronic health or mhealth or m-health or mobile health or virtual health or digital health or technological aid?).tw. | 34518 |
| 50 | ((blackberr* or black-berr*) adj3 (mobile* or phone* or computer*)).tw. | 34 |
| 51 | (google adj3 phone*).tw. | 17 |
| 52 | exp Africa South of the Sahara/ | 259972 |
| 53 | "Sub-Saharan Africa*".tw. | 27165 |
| 54 | African*.tw. | 194869 |
| 55 | (Angola* or Benin* or Botswana* or "Burkina Faso" or Burundi* or Cameroon* or "Cape Verde*" or "Cabo Verde*" or Chad or Comoros or Congo* or "Ivory Coast" or "Cote d'Ivoire" or Djibouti* or Equatorial Guinea or Eritrea* or Ethiopia* or Gabon* or Gambia* or Guinea* or Kenya* or Lesotho or Liberia* or Madagasca* or Malawi* or Mali or Mauritania* or Mauriti* or Mozambi* or Namib* or Niger* or Nigeria* or Rwanda* or "Sao Tome*" or Principe* or Senegal* or Seychelles or "Sierra Leone" or Somali* or South Africa or Sudan* or Swazi* or Eswantini* or Tanzania* or Togo* or Uganda* or Western Sahara or Zambia* or Zimbabw*).tw. | 430992 |
| 56 | 1 or 2 or 3 or 4 or 5 or 6 or 7 or 8 | 542855 |
| 57 | 9 or 10 or 11 or 12 or 13 or 14 or 15 or 16 or 17 or 18 or 19 or 20 or 21 or 22 or 23 or 24 or 25 or 26 or 27 or 28 or 29 or 30 or 31 or 32 or 33 or 34 or 35 or 36 or 37 or 38 or 39 or 40 or 41 or 42 or 43 or 44 or 45 or 46 or 47 or 48 or 49 or 50 or 51 | 379409 |
| 58 | 52 or 53 or 54 or 55 | 645552 |
| 59 | 56 and 57 and 58 | 1608 |

**Database(s): APA PsycInfo 1806 to May Week 3 2020
Search Strategy:**

| **#** | **Searches** | **Results** |
| --- | --- | --- |
| 1 | exp HIV-2/ or exp HIV/ or exp HIV-1/ | 42765 |
| 2 | exp Acquired Immunodeficiency Syndrome/ | 0 |
| 3 | exp hiv/ or exp hiv infections/ | 42765 |
| 4 | (human immunodeficiency or human immunodeficiency virus or human immuno-deficiency virus or human immune-deficiency virus).tw. | 6334 |
| 5 | (hiv or hiv-1* or hiv-2* or hiv1 or hiv2 or human immunodeficiency virus or human immunedeficiency virus or human immuno-deficiency virus or human immune-deficiency virus or (human immun* and deficiency virus) or acquired immunedeficiency syndrome or acquired immuno-deficiency syndrome or acquired immune-deficiency syndrome or (acquired immun* and deficiency syndrome)).tw. | 54619 |
| 6 | HIV.tw. | 52568 |
| 7 | (Immun* adj3 deficienc* adj3 virus*).tw. | 253 |
| 8 | Acquired Immunodeficienc*.tw. | 807 |
| 9 | exp Electronic Health Records/ | 810 |
| 10 | exp eHealth/ | 0 |
| 11 | exp mHealth/ | 922 |
| 12 | exp Internet/ | 29243 |
| 13 | exp Microcomputers/ | 1545 |
| 14 | exp Therapy, Computer-Assisted/ | 0 |
| 15 | exp Cell Phones/ | 5224 |
| 16 | exp Smartphone/ | 0 |
| 17 | exp MP3-Player/ | 0 |
| 18 | exp Computers, Handheld/ | 0 |
| 19 | exp Telemedicine/ | 8608 |
| 20 | exp Videoconferencing/ or Webcasts/ | 561 |
| 21 | exp Text Messaging/ | 928 |
| 22 | exp Telenursing/ | 0 |
| 23 | exp Mobile Applications/ | 805 |
| 24 | exp Social Media/ | 14108 |
| 25 | exp Reminder Systems/ | 0 |
| 26 | exp Electronic Mail/ | 15663 |
| 27 | exp Multimedia/ | 28685 |
| 28 | exp Hypermedia/ | 671 |
| 29 | exp Blogging/ | 0 |
| 30 | Game.tw. | 32046 |
| 31 | ((cell* or mobile*) adj3 (phone* or telephone* or technolog* or device*)).tw. | 9539 |
| 32 | (smartphone* or smart-phone* or cellphone* or mobiles).tw. | 4631 |
| 33 | ((personal adj3 digital) or (PDA adj3 (device* or assistant*)) or MP3 player* or MP4 player*).tw. | 728 |
| 34 | (samsung or nokia).tw. | 153 |
| 35 | (windows adj3 (mobile* or phone*)).tw. | 12 |
| 36 | Android.tw. | 445 |
| 37 | (ipad* or i-pad* or ipod* or i-pod* or iphone* or i-phone*).tw. | 1686 |
| 38 | (tablet* adj3 (device* or computer*)).tw. | 779 |
| 39 | (mhealth or m-health or "mobile health" or ehealth or e-health or "electronic health").tw. | 5002 |
| 40 | (telemedicine or tele-medicine or telehealth or tele-health or telecare or tele-care or telenursing or tele-nursing or telepsychiatry or tele-psychiatry or telemonitor* or tele-monitor* or teleconsult* or tele-consult* or telecounsel* or tele-counsel* or telecoach* or telecoach*).tw. | 4056 |
| 41 | (videoconferenc* or video-conferenc* or webcast* or web-cast*).tw. | 1961 |
| 42 | (((text* or short or voice or multimedia or multi-media or electronic or instant) adj3 messag*) or instant messenger).tw. | 3972 |
| 43 | (texting or texted or texter* or ((sms or mms) adj (service* or messag*)) or interactive voice response* or IVR or voice call* or callback* or voice over internet or VOIP).tw. | 1656 |
| 44 | (Facebook or Twitter or Whatsapp* or Skyp* or YouTube or "You Tube" or Google Hangout*).tw. | 8487 |
| 45 | "mobile app*".tw. | 1456 |
| 46 | (social adj3 (media or network*)).tw. | 41276 |
| 47 | (remind* adj3 (text* or system* or messag*)).tw. | 462 |
| 48 | (electronic mail* or email* or e-mail or webmail).tw. | 9046 |
| 49 | (e-BI or e-SBI or ehealth or e-health or electronic health or mhealth or m-health or mobile health or virtual health or digital health or technological aid?).tw. | 5314 |
| 50 | ((blackberr* or black-berr*) adj3 (mobile* or phone* or computer*)).tw. | 12 |
| 51 | (google adj3 phone*).tw. | 6 |
| 52 | exp Africa South of the Sahara/ | 0 |
| 53 | "Sub-Saharan Africa*".tw. | 4032 |
| 54 | African*.tw. | 66607 |
| 55 | (Angola* or Benin* or Botswana* or "Burkina Faso" or Burundi* or Cameroon* or "Cape Verde*" or "Cabo Verde*" or Chad or Comoros or Congo* or "Ivory Coast" or "Cote d'Ivoire" or Djibouti* or Equatorial Guinea or Eritrea* or Ethiopia* or Gabon* or Gambia* or Guinea* or Kenya* or Lesotho or Liberia* or Madagasca* or Malawi* or Mali or Mauritania* or Mauriti* or Mozambi* or Namib* or Niger* or Nigeria* or Rwanda* or "Sao Tome*" or Principe* or Senegal* or Seychelles or "Sierra Leone" or Somali* or South Africa or Sudan* or Swazi* or Eswantini* or Tanzania* or Togo* or Uganda* or Western Sahara or Zambia* or Zimbabw*).tw. | 44033 |
| 56 | 1 or 2 or 3 or 4 or 5 or 6 or 7 or 8 | 57135 |
| 57 | 9 or 10 or 11 or 12 or 13 or 14 or 15 or 16 or 17 or 18 or 19 or 20 or 21 or 22 or 23 or 24 or 25 or 26 or 27 or 28 or 29 or 30 or 31 or 32 or 33 or 34 or 35 or 36 or 37 or 38 or 39 or 40 or 41 or 42 or 43 or 44 or 45 or 46 or 47 or 48 or 49 or 50 or 51 | 170242 |
| 58 | 52 or 53 or 54 or 55 | 103110 |
| 59 | 56 and 57 and 58 | 577 |

**Database(s): Global Health 1910 to 2020 Week 19
Search Strategy:**

| **#** | **Searches** | **Results** |
| --- | --- | --- |
| 1 | exp HIV-2/ or exp HIV/ or exp HIV-1/ | 178016 |
| 2 | exp Acquired Immunodeficiency Syndrome/ | 0 |
| 3 | exp hiv/ or exp hiv infections/ | 177876 |
| 4 | (human immunodeficiency or human immunodeficiency virus or human immuno-deficiency virus or human immune-deficiency virus).tw. | 173350 |
| 5 | (hiv or hiv-1* or hiv-2* or hiv1 or hiv2 or human immunodeficiency virus or human immunedeficiency virus or human immuno-deficiency virus or human immune-deficiency virus or (human immun* and deficiency virus) or acquired immunedeficiency syndrome or acquired immuno-deficiency syndrome or acquired immune-deficiency syndrome or (acquired immun* and deficiency syndrome)).tw. | 191122 |
| 6 | HIV.tw. | 176728 |
| 7 | (Immun* adj3 deficienc* adj3 virus*).tw. | 1153 |
| 8 | Acquired Immunodeficienc*.tw. | 5070 |
| 9 | exp Electronic Health Records/ | 0 |
| 10 | exp eHealth/ | 0 |
| 11 | exp mHealth/ | 0 |
| 12 | exp Internet/ | 8867 |
| 13 | exp Microcomputers/ | 64 |
| 14 | exp Therapy, Computer-Assisted/ | 0 |
| 15 | exp Cell Phones/ | 2553 |
| 16 | exp Smartphone/ | 2553 |
| 17 | exp MP3-Player/ | 0 |
| 18 | exp Computers, Handheld/ | 0 |
| 19 | exp Telemedicine/ | 1257 |
| 20 | exp Videoconferencing/ or Webcasts/ | 0 |
| 21 | exp Text Messaging/ | 0 |
| 22 | exp Telenursing/ | 0 |
| 23 | exp Mobile Applications/ | 0 |
| 24 | exp Social Media/ | 1840 |
| 25 | exp Reminder Systems/ | 0 |
| 26 | exp Electronic Mail/ | 0 |
| 27 | exp Multimedia/ | 74 |
| 28 | exp Hypermedia/ | 0 |
| 29 | exp Blogging/ | 0 |
| 30 | Game.tw. | 5665 |
| 31 | ((cell* or mobile*) adj3 (phone* or telephone* or technolog* or device*)).tw. | 4703 |
| 32 | (smartphone* or smart-phone* or cellphone* or mobiles).tw. | 1437 |
| 33 | ((personal adj3 digital) or (PDA adj3 (device* or assistant*)) or MP3 player* or MP4 player*).tw. | 178 |
| 34 | (samsung or nokia).tw. | 149 |
| 35 | (windows adj3 (mobile* or phone*)).tw. | 2 |
| 36 | Android.tw. | 553 |
| 37 | (ipad* or i-pad* or ipod* or i-pod* or iphone* or i-phone*).tw. | 355 |
| 38 | (tablet* adj3 (device* or computer*)).tw. | 159 |
| 39 | (mhealth or m-health or "mobile health" or ehealth or e-health or "electronic health").tw. | 3154 |
| 40 | (telemedicine or tele-medicine or telehealth or tele-health or telecare or tele-care or telenursing or tele-nursing or telepsychiatry or tele-psychiatry or telemonitor* or tele-monitor* or teleconsult* or tele-consult* or telecounsel* or tele-counsel* or telecoach* or telecoach*).tw. | 1896 |
| 41 | (videoconferenc* or video-conferenc* or webcast* or web-cast*).tw. | 272 |
| 42 | (((text* or short or voice or multimedia or multi-media or electronic or instant) adj3 messag*) or instant messenger).tw. | 1630 |
| 43 | (texting or texted or texter* or ((sms or mms) adj (service* or messag*)) or interactive voice response* or IVR or voice call* or callback* or voice over internet or VOIP).tw. | 521 |
| 44 | (Facebook or Twitter or Whatsapp* or Skyp* or YouTube or "You Tube" or Google Hangout*).tw. | 1087 |
| 45 | "mobile app*".tw. | 516 |
| 46 | (social adj3 (media or network*)).tw. | 6534 |
| 47 | (remind* adj3 (text* or system* or messag*)).tw. | 494 |
| 48 | (electronic mail* or email* or e-mail or webmail).tw. | 2059 |
| 49 | (e-BI or e-SBI or ehealth or e-health or electronic health or mhealth or m-health or mobile health or virtual health or digital health or technological aid?).tw. | 3442 |
| 50 | ((blackberr* or black-berr*) adj3 (mobile* or phone* or computer*)).tw. | 2 |
| 51 | (google adj3 phone*).tw. | 2 |
| 52 | exp Africa South of the Sahara/ | 212857 |
| 53 | "Sub-Saharan Africa*".tw. | 15385 |
| 54 | African*.tw. | 75563 |
| 55 | (Angola* or Benin* or Botswana* or "Burkina Faso" or Burundi* or Cameroon* or "Cape Verde*" or "Cabo Verde*" or Chad or Comoros or Congo* or "Ivory Coast" or "Cote d'Ivoire" or Djibouti* or Equatorial Guinea or Eritrea* or Ethiopia* or Gabon* or Gambia* or Guinea* or Kenya* or Lesotho or Liberia* or Madagasca* or Malawi* or Mali or Mauritania* or Mauriti* or Mozambi* or Namib* or Niger* or Nigeria* or Rwanda* or "Sao Tome*" or Principe* or Senegal* or Seychelles or "Sierra Leone" or Somali* or South Africa or Sudan* or Swazi* or Eswantini* or Tanzania* or Togo* or Uganda* or Western Sahara or Zambia* or Zimbabw*).tw. | 264960 |
| 56 | 1 or 2 or 3 or 4 or 5 or 6 or 7 or 8 | 191687 |
| 57 | 9 or 10 or 11 or 12 or 13 or 14 or 15 or 16 or 17 or 18 or 19 or 20 or 21 or 22 or 23 or 24 or 25 or 26 or 27 or 28 or 29 or 30 or 31 or 32 or 33 or 34 or 35 or 36 or 37 or 38 or 39 or 40 or 41 or 42 or 43 or 44 or 45 or 46 or 47 or 48 or 49 or 50 or 51 | 32123 |
| 58 | 52 or 53 or 54 or 55 | 320532 |
| 59 | 56 and 57 and 58 | 736 |

## **Supplementary Materials 2**: Data extraction spreadsheet

Please see tab 1 of accompanying spreadsheet.

##

## **Supplementary Materials 3**: TIDieR Checklist

Please see tab 2 of accompanying spreadsheet.

## **Supplementary Materials 4**: Mixed Methods Appraisal Tool for included studies

Please see tab 3 of accompanying spreadsheet.

## **Supplementary Materials 5**: PRISMA checklist

| **Section/topic** | **#** | | **Checklist item** | | **Reported on page #** |
| --- | --- | --- | --- | --- | --- |
| **TITLE** | | | | |  |
| Title | 1 | | Identify the report as a systematic review, meta-analysis, or both. | | 1 |
| **ABSTRACT** | | | | |  |
| Structured summary | 2 | | Provide a structured summary including, as applicable: background; objectives; data sources; study eligibility criteria, participants, and interventions; study appraisal and synthesis methods; results; limitations; conclusions and implications of key findings; systematic review registration number. | | 2 |
| **INTRODUCTION** | | | | |  |
| Rationale | 3 | | Describe the rationale for the review in the context of what is already known. | | 3 |
| Objectives | 4 | | Provide an explicit statement of questions being addressed with reference to participants, interventions, comparisons, outcomes, and study design (PICOS). | | 4 |
| **METHODS** | | | | |  |
| Protocol and registration | 5 | | Indicate if a review protocol exists, if and where it can be accessed (e.g., Web address), and, if available, provide registration information including registration number. | | 4 |
| Eligibility criteria | 6 | | Specify study characteristics (e.g., PICOS, length of follow-up) and report characteristics (e.g., years considered, language, publication status) used as criteria for eligibility, giving rationale. | | 4 |
| Information sources | 7 | | Describe all information sources (e.g., databases with dates of coverage, contact with study authors to identify additional studies) in the search and date last searched. | | 4-5 |
| Search | 8 | | Present full electronic search strategy for at least one database, including any limits used, such that it could be repeated. | | Supplementary materials |
| Study selection | 9 | | State the process for selecting studies (i.e., screening, eligibility, included in systematic review, and, if applicable, included in the meta-analysis). | | 5 |
| Data collection process | 10 | | Describe method of data extraction from reports (e.g., piloted forms, independently, in duplicate) and any processes for obtaining and confirming data from investigators. | | 5 |
| Data items | 11 | | List and define all variables for which data were sought (e.g., PICOS, funding sources) and any assumptions and simplifications made. | | 4-5 |
| Risk of bias in individual studies | 12 | | Describe methods used for assessing risk of bias of individual studies (including specification of whether this was done at the study or outcome level), and how this information is to be used in any data synthesis. | | 5 |
| Summary measures | 13 | | State the principal summary measures (e.g., risk ratio, difference in means). | | N/A |
| Synthesis of results | 14 | | Describe the methods of handling data and combining results of studies, if done, including measures of consistency (e.g., I^2^) for each meta-analysis. | | N/A |
| Risk of bias across studies | | 15 | | Specify any assessment of risk of bias that may affect the cumulative evidence (e.g., publication bias, selective reporting within studies). | N/A |
| Additional analyses | | 16 | | Describe methods of additional analyses (e.g., sensitivity or subgroup analyses, meta-regression), if done, indicating which were pre-specified. | N/A |
| **RESULTS** | | | | |  |
| Study selection | | 17 | | Give numbers of studies screened, assessed for eligibility, and included in the review, with reasons for exclusions at each stage, ideally with a flow diagram. | 6 |
| Study characteristics | | 18 | | For each study, present characteristics for which data were extracted (e.g., study size, PICOS, follow-up period) and provide the citations. | 6-9 |
| Risk of bias within studies | | 19 | | Present data on risk of bias of each study and, if available, any outcome level assessment (see item 12). | N/A |
| Results of individual studies | | 20 | | For all outcomes considered (benefits or harms), present, for each study: (a) simple summary data for each intervention group (b) effect estimates and confidence intervals, ideally with a forest plot. | 23-34 |
| Synthesis of results | | 21 | | Present results of each meta-analysis done, including confidence intervals and measures of consistency. | N/A |
| Risk of bias across studies | | 22 | | Present results of any assessment of risk of bias across studies (see Item 15). | 7 |
| Additional analysis | | 23 | | Give results of additional analyses, if done (e.g., sensitivity or subgroup analyses, meta-regression [see Item 16]). | N/A |
| **DISCUSSION** | | | | |  |
| Summary of evidence | | 24 | | Summarize the main findings including the strength of evidence for each main outcome; consider their relevance to key groups (e.g., healthcare providers, users, and policy makers). | 9-12 |
| Limitations | | 25 | | Discuss limitations at study and outcome level (e.g., risk of bias), and at review-level (e.g., incomplete retrieval of identified research, reporting bias). | 11-12 |
| Conclusions | | 26 | | Provide a general interpretation of the results in the context of other evidence, and implications for future research. | 10-11 |
| **FUNDING** | | | | |  |
| Funding | | 27 | | Describe sources of funding for the systematic review and other support (e.g., supply of data); role of funders for the systematic review. | 13 |
